# Cell-GraphCompass: modeling single cells with graph structure foundation model

**DOI:** 10.1093/nsr/nwaf255

**Published:** 2025-06-24

**Authors:** Chen Fang, Wentao Cui, Zhilong Hu, Wenhao Liu, Shubai Chen, Shaole Chang, Qingqing Long, Cong Li, Yana Liu, Haiping Jiang, Pengfei Wang, Jia Pan, Guoping Hu, Guole Liu, Zhen Meng, Yuanchun Zhou, Linghui Chen, Guihai Feng, Xin Li

**Affiliations:** School of Advanced Interdisciplinary Sciences, University of Chinese Academy of Sciences, Beijing 100049, China; State Key Laboratory of Organ Regeneration and Reconstruction, Institute of Zoology, Chinese Academy of Sciences, Beijing 100101, China; Beijing Institute for Stem Cell and Regenerative Medicine, Beijing 100101, China; Computer Network Information Center, Chinese Academy of Sciences, Beijing 100083, China; iFLYTEK Research, Hefei 230088, China; Computer Network Information Center, Chinese Academy of Sciences, Beijing 100083, China; University of Chinese Academy of Sciences, Beijing 100049, China; Computer Network Information Center, Chinese Academy of Sciences, Beijing 100083, China; iFLYTEK Research, Hefei 230088, China; University of Chinese Academy of Sciences, Beijing 100049, China; State Key Laboratory of Organ Regeneration and Reconstruction, Institute of Zoology, Chinese Academy of Sciences, Beijing 100101, China; Beijing Institute for Stem Cell and Regenerative Medicine, Beijing 100101, China; Institute of Computing Technology, Chinese Academy of Sciences, Beijing 100190, China; University of Chinese Academy of Sciences, Beijing 100049, China; iFLYTEK Research, Hefei 230088, China; Computer Network Information Center, Chinese Academy of Sciences, Beijing 100083, China; University of Chinese Academy of Sciences, Beijing 100049, China; State Key Laboratory of Organ Regeneration and Reconstruction, Institute of Zoology, Chinese Academy of Sciences, Beijing 100101, China; Beijing Institute for Stem Cell and Regenerative Medicine, Beijing 100101, China; University of Chinese Academy of Sciences, Beijing 100049, China; State Key Laboratory of Organ Regeneration and Reconstruction, Institute of Zoology, Chinese Academy of Sciences, Beijing 100101, China; Beijing Institute for Stem Cell and Regenerative Medicine, Beijing 100101, China; State Key Laboratory of Organ Regeneration and Reconstruction, Institute of Zoology, Chinese Academy of Sciences, Beijing 100101, China; Beijing Institute for Stem Cell and Regenerative Medicine, Beijing 100101, China; Computer Network Information Center, Chinese Academy of Sciences, Beijing 100083, China; University of Chinese Academy of Sciences, Beijing 100049, China; iFLYTEK Research, Hefei 230088, China; iFLYTEK Research, Hefei 230088, China; State Key Laboratory of Organ Regeneration and Reconstruction, Institute of Zoology, Chinese Academy of Sciences, Beijing 100101, China; Beijing Institute for Stem Cell and Regenerative Medicine, Beijing 100101, China; Human Organ Physiopathology Emulation System, Institute of Zoology, Chinese Academy of Sciences, Beijing 100101, China; University of Chinese Academy of Sciences, Beijing 100049, China; Computer Network Information Center, Chinese Academy of Sciences, Beijing 100083, China; University of Chinese Academy of Sciences, Beijing 100049, China; School of Advanced Interdisciplinary Sciences, University of Chinese Academy of Sciences, Beijing 100049, China; Computer Network Information Center, Chinese Academy of Sciences, Beijing 100083, China; Oristruct Biotech Co., Ltd, Hefei 230026, China; State Key Laboratory of Organ Regeneration and Reconstruction, Institute of Zoology, Chinese Academy of Sciences, Beijing 100101, China; Beijing Institute for Stem Cell and Regenerative Medicine, Beijing 100101, China; Human Organ Physiopathology Emulation System, Institute of Zoology, Chinese Academy of Sciences, Beijing 100101, China; University of Chinese Academy of Sciences, Beijing 100049, China; School of Advanced Interdisciplinary Sciences, University of Chinese Academy of Sciences, Beijing 100049, China; State Key Laboratory of Organ Regeneration and Reconstruction, Institute of Zoology, Chinese Academy of Sciences, Beijing 100101, China; Beijing Institute for Stem Cell and Regenerative Medicine, Beijing 100101, China; Human Organ Physiopathology Emulation System, Institute of Zoology, Chinese Academy of Sciences, Beijing 100101, China

**Keywords:** single-cell, transcriptomics, foundation model, pre-training, graph neural network, knowledge embedding

## Abstract

Cells in the human body are regulated by sophisticated networks of gene regulation, which allows them to fulfill their cellular destiny and function. Inspired by the advancements in large language models, there have been several attempts focusing on constructing foundation models with single-cell transcriptomic data to decipher gene regulatory networks. However, these models tend to impose a sequential structure on genes within each cell, which may omit intrinsic biological characteristics and lack the utilization of other available prior knowledge. In this paper, we introduce Cell-GraphCompass (CGCompass), the pioneering foundation model that employs graph pre-training to model genes and cells. We use three types of gene-related information as node features for constructing cell graphs and collect data from three perspectives depicting relationships between genes as edge features. We pre-trained the model with over 50 million human cells and then fine-tuned it to a broad spectrum of tasks, such as batch integration, cell type annotation, single-cell gene perturbation and *in silico* gene knockout predictions, achieving commendable performance. Overall, CGCompass provides a practical architecture for leveraging graph pre-training to incorporate prior knowledge in constructing a foundation model for single-cell analysis.

## INTRODUCTION

Investigating the regulatory mechanisms between genes enhances our understanding of biological processes and plays a pivotal role in interpreting cellular functions, treating diseases and promoting biotechnological innovations. Due to the intricate nature of biological networks, compounded by the prohibitive cost of wet-lab experiments, researchers urgently require efficacious computational simulation methods to help decipher gene regulatory mechanisms. Deep learning, at the forefront of artificial intelligence, has emerged as a research hotspot in recent years, thanks to its powerful fitting and predictive capabilities. However, its data-driven essence often constrains its utility in scenarios with limited data. The ‘pre-train and fine-tune’ paradigm [1–4,5], originating from natural language processing (NLP), offers a compelling solution. This kind of approach generally involves pre-training a foundation model on extensive unlabeled datasets, followed by fine-tuning on small, task-specific datasets, thereby transferring generalized insights to domain-specific expertise. In parallel, the advancements in single-cell sequencing technologies provide high-resolution tools for elucidating gene regulatory mechanisms along with cellular heterogeneity. Its associated research has accumulated a vast amount of transcriptomic data in recent years, providing a robust data resource for training foundation models.

Recent advancements have pioneered foundation models in the single-cell domain. scBERT [6] utilized the BERT [2] architecture to develop the first pre-trained model for cell-type annotation. Geneformer [7], scGPT [8] and scFoundation [9] introduced transfer learning to address data limitations in the single-cell field, while GeneCompass [10] achieved breakthroughs across species. These advancements push the boundaries of single-cell analysis and they are all based on the transformer architecture [1], which was primarily designed for handling textual sequence data within the NLP domain. To meet the input demands of transformers, cells are conceptualized as ‘sentences’ and genes as ‘words’. Nevertheless, unlike text, there is no inherent sequential structure among genes within cells, so modeling them as graph structures may be more appropriate. Moreover, previous work has focused exclusively on extracting information from transcriptional expression profiles, neglecting the wealth of biological prior knowledge and previous research findings. Integrating prior knowledge from multiscale biological processes may not only enhance the model's performance across different downstream applications but also reduce the model's dependence on the quality of collected sequencing data.

In this paper, we introduce Cell-GraphCompass (CGCompass), a graph-based, knowledge-guided foundation model pre-trained on large-scale single-cell sequencing data. CGCompass conceptualizes each cell as a graph, with nodes representing the genes it contains and edges denoting the relationships between them. We employ a graph neural network [11,12,13] (GNN)-based architecture that integrates six different types of features and utilizes the message-passing mechanisms along with self-attention mechanisms to jointly learn the embedding representations of genes and cells. Our model was pre-trained on 50 million human single-cell sequencing data from scCompass-h50M [14] and validated its effectiveness across a broad range of downstream tasks. Both gene-level and cell-level zero-shot experiments demonstrated that CGCompass, through pre-training, could learn meaningful biological knowledge and generate insightful gene and cell representations. CGCompass was also adopted for various downstream tasks through fine-tuning, including batch integration, cell type annotation, single-cell gene perturbation and *in silico* gene knockout prediction. Extensive comparison and ablation experiments demonstrated the capability of CGCompass across these application scenarios.

## RESULTS

### The graph-based foundation model overview

CGCompass is a single-cell foundation model based on GNN and transformer architecture. We incorporate various biological features and previously summarized knowledge to provide the model with prior information. We collected over 50 million human single-cell sequencing data for pre-training, equipping CGCompass with general knowledge and generalization capability.

**Figure 1.**
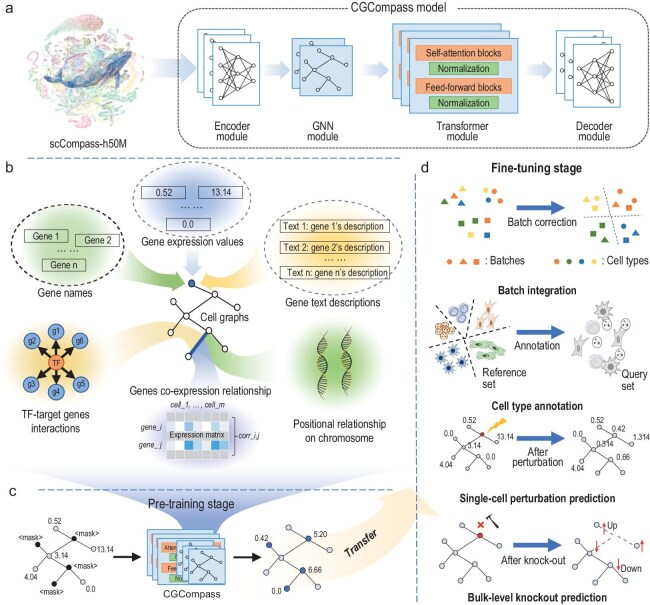
Overview of CGCompass methodology. (a) Model architecture: CGCompass consists of four components: an encoder, a GNN, a transformer and a decoder module. (b) Biological features used to build the cell graph: CGCompass utilizes gene names, gene transcription expression values, and gene text descriptions as node features and constructs edges based on TF–TG interactions, gene co-expression relationships, and positional relationship of genes on chromosome. (c) Pre-training stage: CGCompass learns general knowledge about genes through masked training on scCompass-h50M. (d) Downstream applications: CGCompass reaches state-of-the-art outcomes in a plethora of downstream tasks, exemplified by four instances in this paper: batch integration; cell type annotation; single-cell gene perturbation prediction; and bulk knockout prediction.

The CGCompass model architecture consists of four components (Fig. 1a): an encoder module, a GNN module, a transformer module and a decoder module. The encoder module digitizes different forms of input features for GNN processing. The GNN module uses a message-passing mechanism to propagate gene feature information between connected nodes, enabling local feature extraction. The transformer module captures long-range associations of genes on the cell graph using self-attention mechanisms, compensating for the GNN's lack of a global perspective. Together, the GNN and transformer modules provide context-aware gene embeddings for the input single-cell sequencing data. The decoder module converts these gene embeddings into the desired output format based on specific application scenarios.

To construct the cell graph, we used six types of feature data to describe genes and their interactions from different aspects (Fig. 1b). The transcriptional expression of genes within a cell can be seen as a snapshot of the cell's state and context, so we use gene names and their transcriptional expression in the cell as two node features of the cell graph. Additionally, extensive existing gene research is documented. To fully utilize this, we use the pre-trained language model BioBERT [15], designed for biomedicine, to extract a third node feature from gene-related literature [16]. For building the edges of the cell graph, we collected three types of data describing the relationships between genes: regulatory relationships between transcription factors and their target genes; statistically quantified gene co-expression patterns; and the positional information of genes on chromosomes (see Supplementary Methods). These edges indicate the direction of message passing in the GNN and enrich the model's understanding of gene relationships.

After building the model and input cell graph, we conducted large-scale pre-training and various downstream tasks for CGCompass. For pre-training, we utilized approximately 50 million human single-cell transcriptomes from scCompass-h50M and designed a unified data processing workflow. Our pre-training strategy randomly masks 40% of gene expression values for each input and uses the remaining 60% to infer the overall cellular state and predict the expression of the masked genes (Fig. 1c). Additionally, a global node representing the entire cell was introduced and connected to all gene-specific nodes, explicitly modeling the cellular state. After pre-training, CGCompass can be fine-tuned for different downstream tasks, such as batch integration, cell type annotation, single-cell gene perturbation and *in silico* gene knockout prediction (Fig. 1d). Its flexible modular design allows for simple adjustments to accommodate different application scenarios, making it adaptable for fine-tuning to address specific problems.

### Through pre-training, CGCompass acquires biologically meaningful knowledge

After pre-training on large-scale single-cell RNA sequencing data, CGCompass theoretically possesses the capability to understand the functions and characteristics of most genes and to develop a general understanding of gene interactions within single cells. To test whether CGCompass truly possesses this capability and can further transfer to specific problem scenarios without fine-tuning like large language models (LLMs), we evaluated its generalization ability in a zero-shot manner using tasks within different biological backgrounds. Following pre-training, the embeddings stored in the CGCompass vocabulary encapsulate the model's overall understanding of each gene (see Supplementary Methods), so we used them as the basis for all testing experiments in this session.

**Figure 2.**
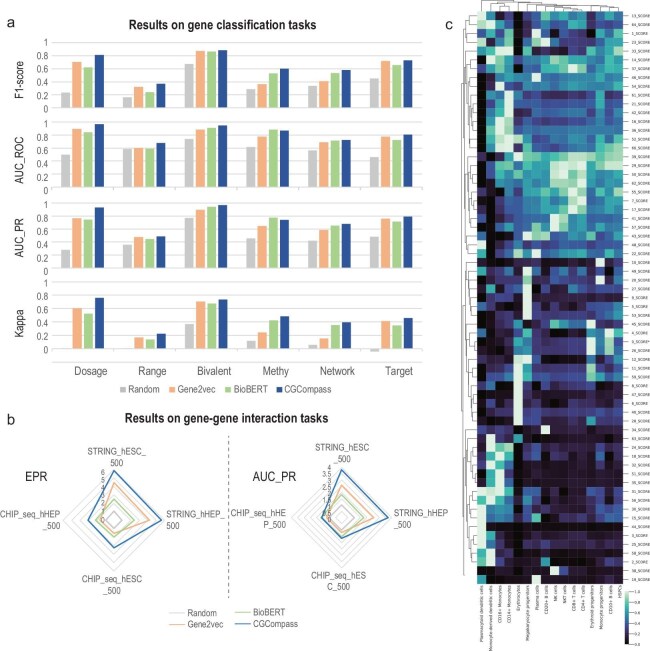
Pre-training endows CGCompass with biologically meaningful knowledge. By dissecting the latent space of the pre-trained model, we tested whether CGCompass can, to some extent, identify genes and the interaction between them. We extracted the gene embeddings from CGCompass vocabulary and used them for gene-level classification tasks and GGI prediction. (a) Results of six gene classification experiments. The *x*-axis represents six different datasets, while the *y*-axis corresponds to four metrics. The value range for all metrics is between 0 and 1, with higher values indicating better performance. (b) Results of GGI experiments. The four axes of the radar plot represent four different datasets. The early precision ratio (EPR) metric measures the overlap between the model-reconstructed GGI and the ground truth. The area under the precision-recall curve (AUC_PR) metric evaluates the extent to which the model predicts the ground truth GGI. (c) The selective activation of the gene program extracted by CGCompass across different cell types. Detailed label information can be found in Fig. S2.

To assess whether CGCompass can recognize specific genes, we selected six binary classification datasets describing gene functions or characteristics across various aspects, including gene dosage sensitivity, chromatin dynamics and network dynamics. We conducted comparative experiments with three other methods for generating general gene embeddings: gene2vec [17], which produces embeddings based on gene co-expression patterns; BioBERT [15], which generates embeddings from literature descriptions of genes; and randomly generated gene embeddings. We evaluated performance using MacroF1, AUC_PRC, AUC_PR and the kappa coefficient as metrics (see Supplementary Methods). Results of the 5-fold cross-validation (Fig. 2a, Fig. S1a–f and Tables S1–S6) showed that the embeddings generated by the three test models significantly outperformed those produced randomly, indicating that these embeddings captured gene-related information to varying degrees. Moreover, CGCompass achieved the best results across all tasks, demonstrating it could learn meaningful gene-related knowledge through pre-training.

Next, we evaluated CGCompass's ability to understand gene relationships. We used eight datasets from the STRING [18] and ChIP-seq [19–21] platforms to conduct gene–gene interaction (GGI) prediction experiments. Following the methodology of DeepSEM [22], we assessed the potential interactions by calculating the cosine similarity of normalized gene embeddings. The quality of interactions predicted by CGCompass and three other methods was evaluated using the EPR and AUC_PR metrics (Supplementary Methods). Results across all eight datasets showed that CGCompass embeddings consistently reconstructed the most comprehensive GGI relationships (Fig. 2b and Fig. S1g), demonstrating that pre-training allowed CGCompass to effectively learn gene interactions.

Gene programs can also serve as a means to evaluate the quality of gene embeddings. We clustered genes based on their embeddings, with each cluster representing a potential gene program. We then analyzed the expression patterns of these gene programs in a human immune tissue dataset [23]. As shown in Fig. 2c and Fig. S2, functionally similar genes were grouped into the same program. Each gene program exhibited selective expression in specific cell types, demonstrating that CGCompass can identify biologically meaningful functional gene clusters.

In summary, we extracted the embeddings of CGCompass's gene vocabulary and tested its performance in gene classification, gene interaction prediction and gene program extraction tasks. The experimental results show that the pre-training of CGCompass can, to some extent, acquire meaningful biological knowledge. However, the vocabulary embeddings do not involve cell context information. To test whether CGCompass can correctly capture context-aware information and use it to solve practical problems, the following four chapters will fine-tune CGCompass to solve specific downstream tasks.

### Integrating multi-batch single-cell sequencing data with CGCompass

In real-world scenarios, single-cell RNA-seq datasets are often derived from different experimental instruments, sequencing platforms and analysis methods. These variations introduce technical noise [24], complicating the extraction of meaningful biological insights. Therefore, we evaluated CGCompass's performance on batch integration tasks, conducting both fine-tuning and zero-shot experiments to access its ability to accurately and consistently capture cell type-related information across batches of data.

**Figure 3.**
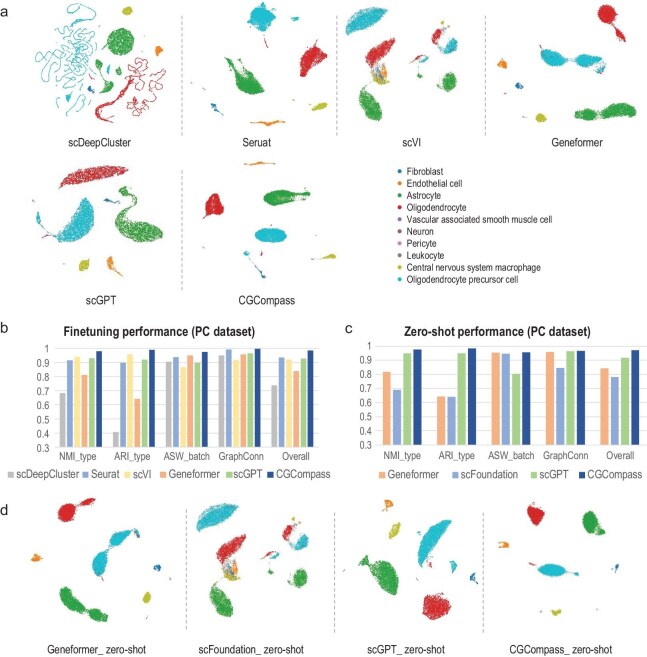
Integrating multiple batches of data with CGCompass. On the PC dataset, (a) UMAP plots of cell embeddings generated by fine-tuned CGCompass and other baseline models, colored by the ground truth cell types. (b) Quantitative metric results of fine-tuned CGCompass and other baseline models. The value range for all metrics is between 0 and 1, with higher values indicating better performance. (c) Quantitative metric results of different foundation models’ zero-shot performance. (d) UMAP plots of zero-shot embeddings from foundation models.

We first conducted fine-tuning experiments on a perirhinal cortex (PC) dataset [25], which contains sequencing data from 10 cell types across two batches. CGCompass was fine-tuned on the PC dataset using the same self-supervised learning method as in the pre-training process, converging from a general model to a PC-specific model. We used four quantitative metrics to evaluate clustering results: NMI; ARI; ASW; and GraphConn, with their average results serving as the overall score (see Supplementary Methods). The first two metrics assess clustering results by cell types, reflecting the model's capacity to retain biological signals, while the latter two measure the mixing of data from different batches, reflecting the ability to mitigate batch variations. We compared CGCompass against three models [26–28] trained from scratch and two fine-tuned foundation models [7,8]. Uniform manifold approximation and projection (UMAP) dimensionality reduction was applied to cell embeddings generated by each model, and the visualization (Fig. 3a and Fig. S3) demonstrated that CGCompass achieved the best clustering performance, particularly for astrocyte and oligodendrocyte cells. The quantitative metrics further confirmed that CGCompass outperformed the other models (Fig. 3b and Table S7), demonstrating its ability to learn cell type-related information based on gene expression profiles without explicit supervision.

Zero-shot inference represents a distinguishing capability of pre-trained foundation models. Since the model is pre-trained on a diverse set of cell types, it can generate reasonably accurate embeddings for new cells without the need for further fine-tuning. Therefore, we tested CGCompass's zero-shot performance on batch integration tasks against the three leading foundation models [7–9]. CGCompass achieved the highest metric scores (Fig. 3c and d and Table S8), although with a minimal decline in performance compared with its fine-tuned results.

To demonstrate the effectiveness of graph-structured pre-training, we conducted ablation experiments on both the graph structure and the pre-training process. For fair comparison, we first ablated the graph structure and pre-trained a single transformer-based model on scCompass-h50M, then fine-tuned it on the PC dataset using the same loss function. Results (Table S9) showed that our pre-training model based on the cell graph was more effective than the single transformer pre-trained model. Secondly, without changing the model, we ablated the pre-training process, training the model from scratch on the PC dataset and the results (Table S10) showed a marked decrease in performance without pre-training.

We conducted similar fine-tuning and zero-shot experiments on three additional datasets. The PBMC 10k dataset [29] includes data from two sequencing batches, while the COVID-19 [30] and human pancreas [23] datasets are more complex, encompassing 18 and 9 batches, respectively. Clustering visualizations for all three datasets are presented in Figs. S4–S6. In both fine-tuning and zero-shot settings, CGCompass consistently produced high-quality cell embeddings. Clustering evaluation metrics are summarized in Tables S11–S20, where CGCompass consistently outperformed other models, achieving the highest performance. Notably, in the human pancreas dataset, which includes data from multiple sequencing platforms with inherent batch effects, CGCompass effectively mitigated these platform-related discrepancies, yielding robust and accurate cell embeddings.

### CGCompass exhibits commendable performance in cell type annotation

**Figure 4.**
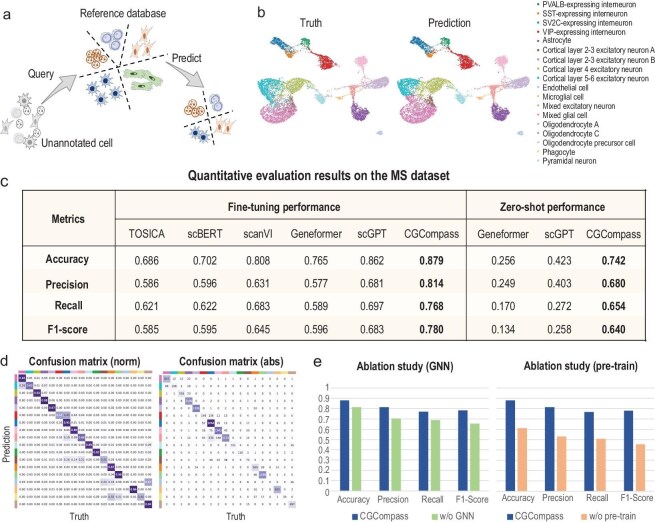
CGCompass boosts cell type annotation. (a) Schematic of the cell type annotation task. On the MS dataset, (b) UMAP visualization of cell embeddings generated by CGCompass, colored by ground truth cell types (left) and CGCompass prediction results (right). (c) Quantitative evaluation of cell annotation by CGCompass and baseline models, with fine-tuning results on the left, and zero-shot results on the right. (d) Confusion matrix between cell types predicted by fine-tuned CGCompass and ground truth labels, with normalization on the left and absolute values on the right. (e) Ablation study on the GNN module and pre-training stage of CGCompass.

After evaluating the performance of CGCompass in batch integration, we proceeded to test its ability for cell type annotation. We provided the model with a reference set to guide it in learning the mapping from RNA-seq inputs to cell type labels, then supplied a separate query set to test the model's annotation performance (Fig. 4a). Unlike batch integration, this is a supervised learning task.

We first conducted experiments on a multiple sclerosis (MS) dataset [31], which includes 17 cell types. The reference set comes from healthy human immune cells and the query set is derived from MS patients. Two models [32,33] trained from scratch and three foundation models [6–8] were chosen for comparison. The predictions of CGCompass are visualized in Fig. 4b, which closely matched the actual distribution of the query set. We then quantitatively assessed the annotation results of each model using common classification metrics (Fig. 4c left and Table S21). It can be observed that CGCompass achieved the best prediction results. Additionally, we analyzed CGCompass's prediction accuracy for each cell type. The confusion matrix (Fig. 4d) indicated that our model achieved over 90% accuracy for most cell types, with only low performance in some very rare types, such as endothelial cells. Ablation studies were also conducted for the cell annotation task (Fig. 4e and Tables S22 and 23), confirming the effectiveness of the graph structure and the necessity of the pre-training process.

Next, we investigated CGCompass's zero-shot annotation capability using a linear probe approach. For each cell in both the reference and query sets, we used CGCompass to generate cell embeddings. A linear model was then trained on the reference embeddings to predict cell types, with its performance evaluated on the query set. The same procedure was applied to test Geneformer and scGPT. As shown in the experimental results (Fig. 4c right and Table S24), only the embeddings generated by CGCompass achieved satisfactory annotation performance. The linear probe results indicate that even without fine-tuning, the embeddings produced by CGCompass contain rich information for cell type prediction.

We further validated CGCompass on three additional public datasets. The myeloid (Mye) [34] reference set comprises 21 cell types from six types of cancer. The query set is from three other types of cancer, including 11 cell types (with experimental results shown in Fig. S7 and Tables S25–S28). In the human pancreas dataset, the reference set was derived from two small datasets of human pancreas cells, encompassing 13 cell types, while the query set came from three other datasets, including 11 cell types [35,36] (with results shown in Fig. S8 and Tables S29–S32). The human lung dataset [19] consists of a reference set from healthy samples, which contains 144 cell subtypes. The query set comes from diseased samples, containing 140 cell subtypes (Tables S33 and S34). These datasets present varying levels of transfer difficulty between the reference and query sets. CGCompass consistently achieved the best annotation results, particularly in the linear probe evaluation.

### CGCompass excels at predicting single-cell gene perturbation response

After exploring cell-level downstream tasks, we now turn our attention to assessing the performance of CGCompass in gene-level application scenarios. Precise prediction of gene perturbation response is crucial for understanding the regulatory patterns among genes. The advancement of gene editing [37] and Perturb-seq [38] technologies has laid the groundwork for the application of deep learning methods [39–41]. GEARS [42] is one of the most advanced perturbation models to date. Similar to our cell graph, it also adopts a GNN-based architecture but utilizes gene co-expression relationships and ontology knowledge to construct graphs.

**Figure 5.**
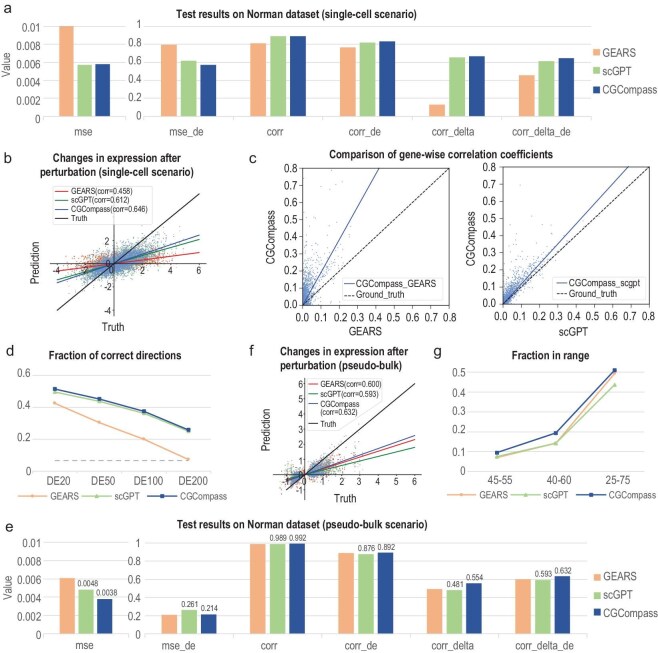
CGCompass predicts single-cell gene perturbation responses accurately. (a) Quantitative comparison of the perturbation predictions between CGCompass and baseline models at single-cell resolution, with lower *mse* and higher *corr* indicating better performance. (b) Scatter plot of all models’ predictions on gene expression changes after perturbation. The *x*-axis represents the true values, and the *y*-axis represents the predicted values, with each point corresponding to a gene in a certain cell, showcasing 1% of total genes. Lines of different colors fit the scatter plots of different models. (c) Comparison of gene-wise correlations between CGCompass and baseline models. Each point represents a gene, with the *y*-axis showing the correlation coefficient between CGCompass's predictions and the ground truth across all cells, and the *x*-axis representing the corresponding results from the baseline model. (d) Accuracy of all models’ predictions on the direction of gene change (increase, no change, decrease), with the *x*-axis representing the different number of DE genes included in the evaluation. (e) Quantitative comparison of the predictions between all models in the pseudo-bulk scenario. (f) Scatter plot of all models’ predictions on the average gene expression changes by perturbation condition. Each point represents the average expression value of a gene under one certain perturbation condition. (g) The predictive accuracy of each model within confidence intervals of various scopes.

Our empirical evaluation began with a public dataset named Norman [43], which includes 105 single-gene and 131 double-genes perturbations. We first assessed CGCompass's prediction performance at single-cell resolution. Following the same dataset split as GEARS, we ensured that the test set contains perturbation conditions (which gene/s are perturbed) not present in the training set. We used GEARS and scGPT as baselines. Evaluation metrics included mean squared error (*mse*) and Pearson correlation coefficient (*corr*) of gene expression values after perturbation, along with the correlation of gene expression change (*corr_delta*). These metrics were accessed across all genes (*_all*) and separately for the top 20 differentially expressed genes (*_de*). As shown in Fig. 5a and b, both foundation models outperform GEARS, highlighting the importance of pre-training in this task. Furthermore, CGCompass surpassed scGPT, demonstrating the benefits of a graph-based foundation model. We also computed per-gene correlation between predicted and actual expression profiles. As shown in Fig. 5c, CGCompass consistently made predictions closer to ground truth for most genes. Additionally, we evaluated the models’ ability to predict the direction of gene expression change (upregulated, unchanged, downregulated). CGCompass achieved the highest accuracy across various sets of differentially expressed genes (Fig. 5d).

To mitigate the impact of CRISPR [37] knockout efficiency and the randomness of single-cell sequencing technology, we also considered the pseudo-bulk scenario. In this setting, all cells subjected to the same perturbation were aggregated and treated as a single pseudo-bulk sample. We integrated the single-cell level predictions generated by CGCompass to construct pseudo-bulk profiles and assessed the average performance across all pseudo-bulk samples. Under this evaluation, CGCompass achieved the highest average performance among all compared models (Fig. 5e and f). We further presented case studies of the top 20 differentially expressed genes influenced by four specific two-gene perturbations (Fig. S9) and measured the prediction accuracy of each model within different confidence intervals (Fig. 5g, Supplementary Methods). CGCompass consistently achieved the best results. Ablation studies confirmed the contributions of both the graph structure and the pre-training process (Fig. S10).

The above pseudo-bulk results were aggregated from single-cell predictions. We then tested a direct pseudo-bulk input setting, where the model was fed average expression profiles directly. Both scGPT and CGCompass showed notable performance gains (Table S36), with CGCompass outperforming all baseline methods (Fig. S11a). Despite being trained on single-cell data, CGCompass generalized effectively to the pseudo-bulk setting, demonstrating the flexibility of graph-based foundation models.

We further validated our findings on two additional single-gene perturbation datasets. The Adamson [44] dataset includes 87 perturbation conditions, while the Dixit [38] dataset is a small-sample scenario containing only 20 perturbations (with results shown in Tables S35 and S36 and Fig. S11b and c). CGCompass achieved the best performance on both datasets, with a large margin on the Dixit dataset. These results highlight the model's strong generalization capabilities, especially in few-shot settings.

Given that different foundation models adopt distinct model architectures and pre-training datasets, we compared Geneformer, scGPT and CGCompass using the same pre-training dataset. We randomly sampled 10% of the data from scCompass-h50M, denoted as scCompass-h5M, and re-trained the three models on this subset. Their fine-tuning performance was then evaluated across three downstream tasks: batch integration; cell type annotation; and single-cell gene perturbation prediction. Using the same pre-training dataset, CGCompass consistently outperformed the other models, underscoring the advantages of the CGCompass architecture (Tables S37–S41).

To better understand the contribution of each biological feature to CGCompass, we separately performed ablation of the four features, excluding gene names and gene expression. After each ablation, CGCompass was re-trained on scCompass-h5M, and fine-tuning experiments were conducted across three downstream tasks. The results indicated that the ablation of each feature negatively impacted model performance, demonstrating that each feature contributes to the model (Tables S42–S45). Moreover, the feature causing the greatest performance decline varied across different datasets, suggesting that the relative contributions of each feature differ depending on the specific task or context.

### Exploring bulk gene knockout prediction with CGCompass

After completing the single-cell perturbation study, we extended our exploration to the bulk level. Single-cell gene perturbation focuses on the impact of perturbing certain genes on individual cells, whereas bulk gene perturbation emphasizes the effects on cell populations, which is significant for research in areas such as organ development and tissue regeneration.

Our methodology employed a two-step transfer strategy. First, we pre-trained CGCompass on 20 million mouse single-cell sequencing data. This was followed by a second pre-training phase on 300 000 mouse bulk sequencing data, enabling the transition from a single-cell foundation model to a bulk-level model. Next, we fine-tuned the model on a dataset of over 3300 mouse bulk gene knockouts to adapt the bulk foundation model into a bulk knockout model (Supplementary Methods).

**Figure 6.**
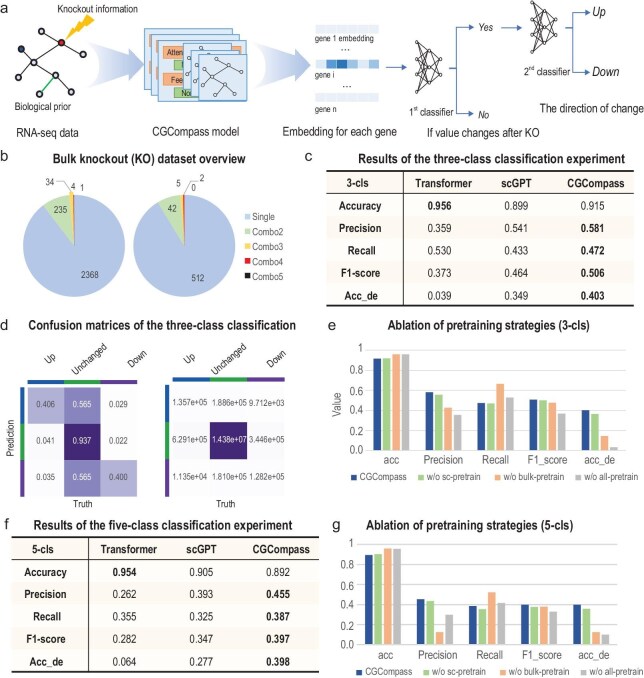
Exploring bulk gene knockout prediction with CGCompass. (a) Knockout model architecture. We designed a ‘two-step’ classifier as the decoder module of CGCompass for the bulk knockout problem scenario. For each gene's embedding, CGCompass first determines whether its expression value changes, then judges whether it is up or down. (b) Distribution of perturbation conditions in the bulk knockout dataset we collected. ‘Single’ represents single-gene knockout, and ‘ComboX’ represents knockout of X genes. (c) In the three-class (up, no change, down) task, classification results of different models. (d) Confusion matrix for CGCompass. (e) Ablation experiment on different pre-training stages. (f) In the five-class (5-fold up, 2-fold up, no change, 2-fold down, 5-fold down) task, classification results of different models. (g) Ablation experiment on different pre-training stages.

We aimed to predict the direction of change for all genes following knockout—categorizing them into three classes: 2-fold upregulation; no change; and 2-fold downregulation. Given the severe class imbalance in the knockout dataset, where upregulated or downregulated genes account for only about 4%, we designed a two-step classifier as the decoder to prioritize the detection of upregulated and downregulated genes (Fig. 6a). For unbiased comparison, we used the encoder from scGPT, paired with our two-step classifier, as the baseline model. Additionally, we implemented a simple transformer encoder followed by our classifier as another baseline. The training and testing datasets include perturbation conditions for both single-gene and multi-gene knockouts (Fig. 6b). The models were evaluated using standard classification metrics, as well as their prediction accuracy for differentially expressed (DE) genes before and after knockout. From the results (Fig. 6c), it is evident that the transformer baseline tends to predict all genes as unchanged, resulting in an overall accuracy as high as 95%, but with near-zero accuracy in detecting DE genes. In contrast, CGCompass outperformed by successfully identifying the highest number of DE genes. To further investigate the potential real-world applicability of our perturbation model, we visualized the confusion matrix of CGCompass's predictions (Fig. 6d). It can be observed that, with an overall accuracy exceeding 90%, nearly half of the genes predicted by CGCompass as upregulated or downregulated were successfully validated. The remainder were mostly unchanged, with fewer than 5% displaying an opposite trend.

Furthermore, to validate the effectiveness of the two-step transfer process, we conducted ablation experiments on both the single-cell pre-training phase and the bulk-level pre-training phase separately. The results (Fig. 6e) indicate that removing either phase leads to a decline in model performance. However, skipping the bulk-level pre-training phase results in a more significant performance drop, underscoring its critical role in the overall effectiveness of the model.

Finally, we refined the granularity of direction prediction, attempting to predict whether a gene is 5-fold upregulated, 2-fold upregulated, unchanged, 2-fold downregulated or 5-fold downregulated. With more specific supervisory signals, CGCompass demonstrated a more significant improvement compared with the three-class classification (Fig. 6f). The results of the confusion matrix (Fig. S10e) and ablation studies (Fig. 6g) were also consistent with the previous conclusions.

## DISCUSSION

In this article, we introduce Cell-GraphCompass, the pioneering single-cell foundation model utilizing graph structures to model genes and cells. Various biological features and prior knowledge were embedded through graph neural networks. The results from zero-shot inference, evaluated at both the gene level and the cell level, confirm that our pre-training successfully captured an understanding of genes and their interactions to a certain extent. The model, after fine-tuning, was used for a diverse range of downstream tasks, achieving a satisfactory level of performance. Batch integration experiments demonstrate CGCompass's effectiveness in overcoming batch effects, while cell type annotation tasks further validate CGCompass's applicability to cell-level problems. Furthermore, we delved into gene perturbation response prediction, where CGCompass improved predictions for single-cell gene perturbations and pioneeringly explored bulk gene knockout prediction, showcasing its potential for practical applications.

Through abundant comparative and ablation studies, we demonstrated the efficacy of the CGCompass methodology. Comparisons with smaller models trained from scratch, along with ablation studies on the pre-training stage, underscored the effectiveness of pre-training operations. Evaluating against other foundation models and ablation of the GNN module confirmed our approach of cell graph modeling. Graph pre-training allows the model to be exposed to various types of cells and associated biological features before being applied to downstream tasks. This accelerates the model's convergence in specific application scenarios and effectively prevents it from converging to local optima. Consequently, it becomes feasible to apply deep learning in many data-sparse situations and alleviates the misleading tendencies caused by biased data.

At the same time, we also recognize that there are still many areas where the model can be improved. The information provided by transcriptomics alone is limited. In the future, a comprehensive multi-omics integrated model could be considered, incorporating data from ATAC-seq, proteomics, epigenomics and other sources. Moreover, with the rapid advancement of LLMs, exploring more advanced LLMs beyond BioBERT for extracting textual information from gene descriptions is promising. GenePT [45] and scGenePT [46] may serve as good examples. Furthermore, to address the issues of data scarcity and imbalance met in downstream tasks, beyond using pre-training and rich biological features, semi-supervised [47] and long-tail [48] learning may offer more possible solutions. Lastly, there remains considerable potential for further improvement in applying CGCompass to specific tasks. CGCompass's graph-based modeling approach emphasizes the integration of multiple biological features, while some other single-cell GNN models [40,42] offer distinct graph construction methodologies and alternative problem-solving strategies.

As foundation models garner increasing attention in the life sciences, some researchers have raised concerns regarding their ability to truly advance scientific discovery in this field [49,50]. In certain application scenarios, foundation models may underperform compared with smaller, task-specific models. It is important to recognize that foundation models are designed as general-purpose frameworks capable of addressing a wide range of downstream tasks. While they may not always outperform models tailored for specific problems, one of their key strengths lies in zero-shot inference—a critical capability in settings where data are extremely limited.

Moreover, the effectiveness of a foundation model often depends on how it is applied. Recently, we noted that some studies have discussed whether linear models or foundation models are better suited for perturbation prediction [49]. When single-cell prediction results are aggregated, foundation models may indeed underperform relative to linear models. However, when alternative approaches are adopted—such as directly inputting pseudo-bulk profiles—the conclusions differ. This observation underscores the importance of continued exploration into how foundation models can be most effectively applied across different biological contexts.

As the ‘pre-training and fine-tuning’ paradigm continues to advance in biology, graph-based foundation models like CGCompass are expected to play an important role by integrating rich biological priors into model training. We also envision these models enabling new directions in areas such as cell fate reprogramming, cancer drug development and organoid culture.

## METHODS

### Constructing cell graphs using biological features

CGCompass abstracts each cell as a topological graph $G = ( {\mathcal{V},\mathcal{E},{\boldsymbol{X}},{\boldsymbol{\ E}}} )$, where the node set $\mathcal{V}$ consists of the genes of interest, typically highly variable genes. The node feature set ${\boldsymbol{X}}$ includes three types of gene-related information: the gene's vocabulary ID; its expression level in the cell; and its textual description from NCBI. The edge set $\mathcal{E}$ and edge feature set ${\boldsymbol{E}}$ include three types of gene–gene relationships we collected: transcription factor (TF)–target gene (TG) interactions; gene co-expression relationships; and the positional information of genes on chromosomes. Detailed descriptions and encoding schemes for all six types of features are provided in Methods S1.

### Assembling and pre-processing pre-training corpus

To support foundation model research, we have constructed a large-scale single-cell transcriptome corpus, scCompass-h50M [10,14]. It contains over 50 million single-cell transcript sequencing entries sourced from various human tissues and organs. The data in scCompass-h50M undergo a unified quality control process to exclude low-quality or damaged cells.

The pre-processing of CGCompass's pre-training data involves normalization, log-transformation, highly variable gene extraction, value binning, tokenization and padding. First, the sequencing counts of all genes within a cell are normalized (with the total sum controlled to 10 000) and log-transformed. Next, the *Seurat*-based algorithm is used to extract 10 000 highly variable genes from each single-cell dataset. From these, 1200 genes are sampled based on their expression values and used as the nodes for the final cell graph. The log-transformed expression values are further processed using value binning (see Supplementary Methods), ultimately forming the transcriptomic features for these nodes. During pre-training, only genes with non-zero expression are used as input nodes, whereas in downstream tasks, highly variable genes are directly used as nodes with their full gene expression values serving as node features.

For the remaining four biological features, we have compiled a comprehensive knowledge base containing information on all genes and gene pairs. Relevant information for the 1200 selected genes is extracted from this knowledge base to form the additional feature inputs for the cell graphs. The knowledge base includes a total of 44 858 types of nodes, 1247 285 types of TF–TG interactions, 2170 758 types of chromatin position relationships, and gene co-expression relationships with a threshold set at 0.6 (i.e. gene pairs with a correlation coefficient greater than 0.6 are connected by an edge).

### Model architecture

The CGCompass model consists of three GNN layers and 12 transformer encoder layers, each with 8 attention heads. The hidden dimension of the model is set to 512. Detailed information on the model architecture and forward propagation equations is provided in Methods S2.

The specifics of CGCompass's pre-training and downstream tasks are described in Methods S3 and S4, respectively. The hyperparameter settings used for model training are listed in Methods S5.

## Supplementary Material

nwaf255_Supplemental_Files

## CODE, DATA AND MATERIALS AVAILABILITY

The code for CGCompass is now open-sourced at GitHub: https://github.com/epang-ucas/Cell-Graph-Compass. The model weights and datasets can be accessed on Zenodo: https://zenodo.org/records/14650474.
