# Supplementary material for: Cell-GraphCompass: modeling single cells with graph structure foundation model
Source: Natl Sci Rev. 2025 Jun 24;12(10):nwaf255. doi: 10.1093/nsr/nwaf255 (PMC12485608; doi:10.1093/nsr/nwaf255)
Supplement: nwaf255_Supplemental_Files [file nwaf255_supplemental_files.zip › Supplementary Figures.docx]

# Supplementary Figures

**
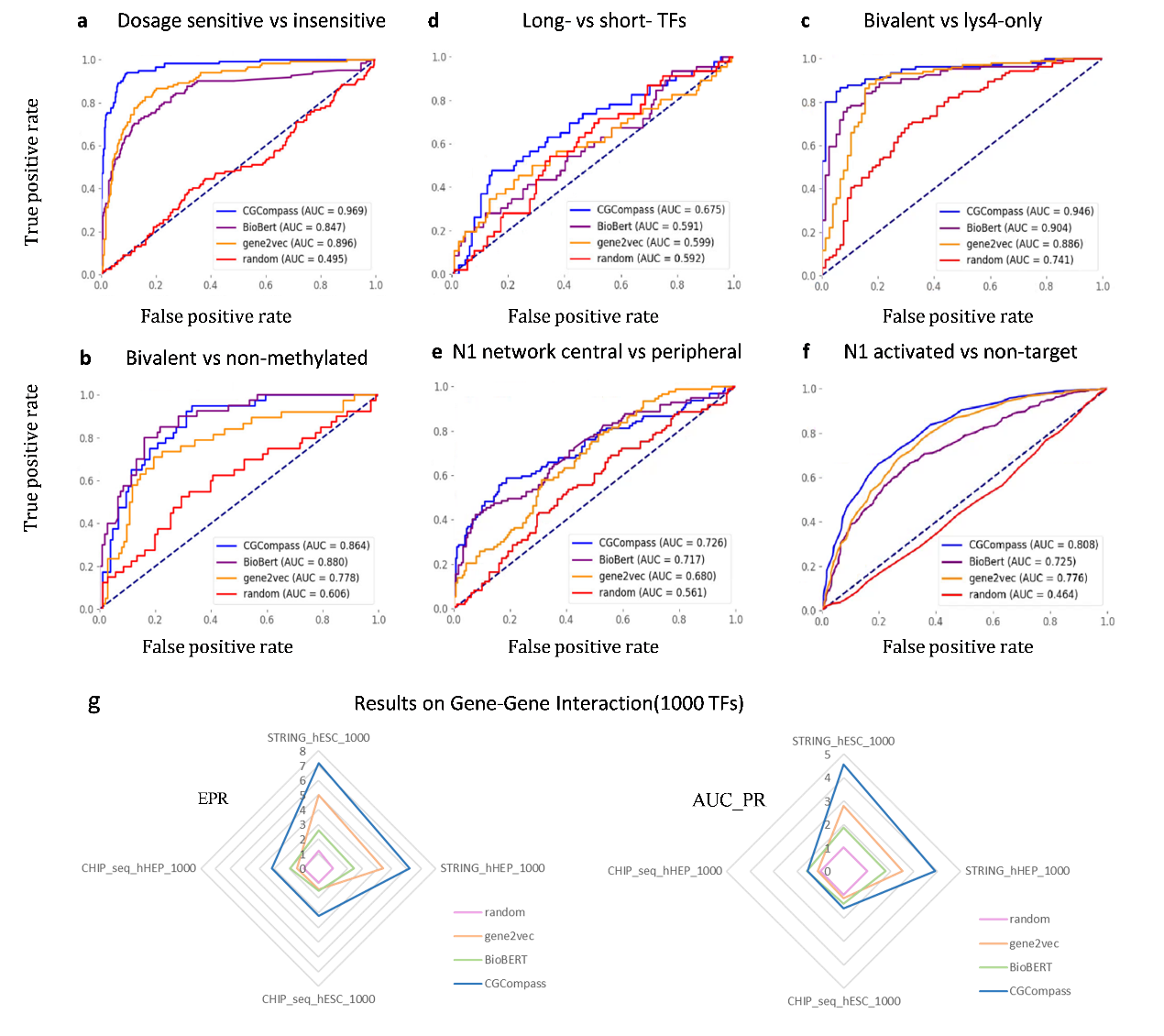
**

**Fig. S**1: a-f, ROC curves of five-fold cross-validation on six gene classification tasks for CGCompass and baseline models. g, Results of the CGCompass and baseline models in the GGI experiments for 1000 TFs, tested from the aspects of EPR and AUC_PR respectively.


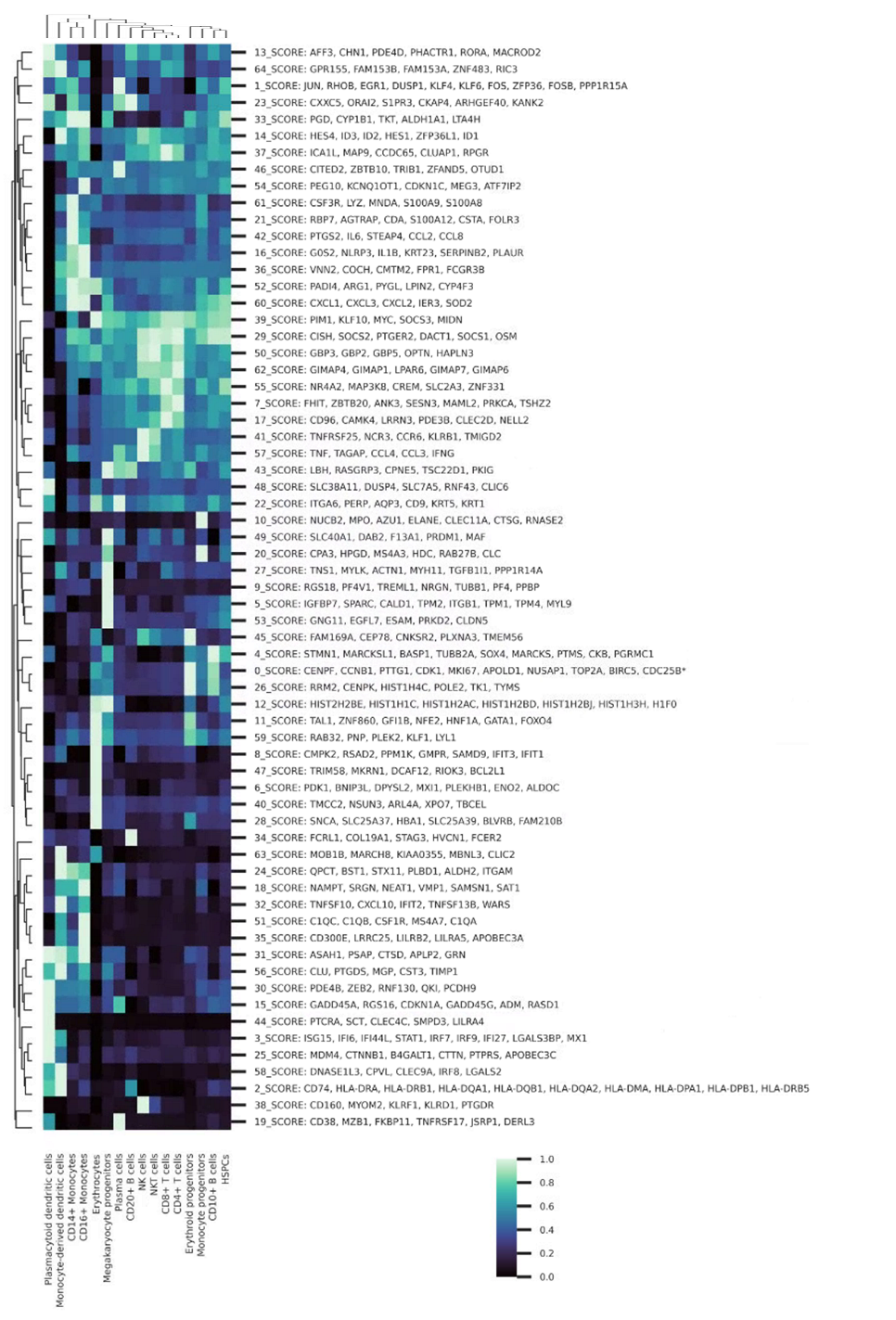


**Fig. S**2: a, Gene programs extracted by CGCompass and their selective expression across different cell types. This figure provides a detailed supplement to Fig. 2d.


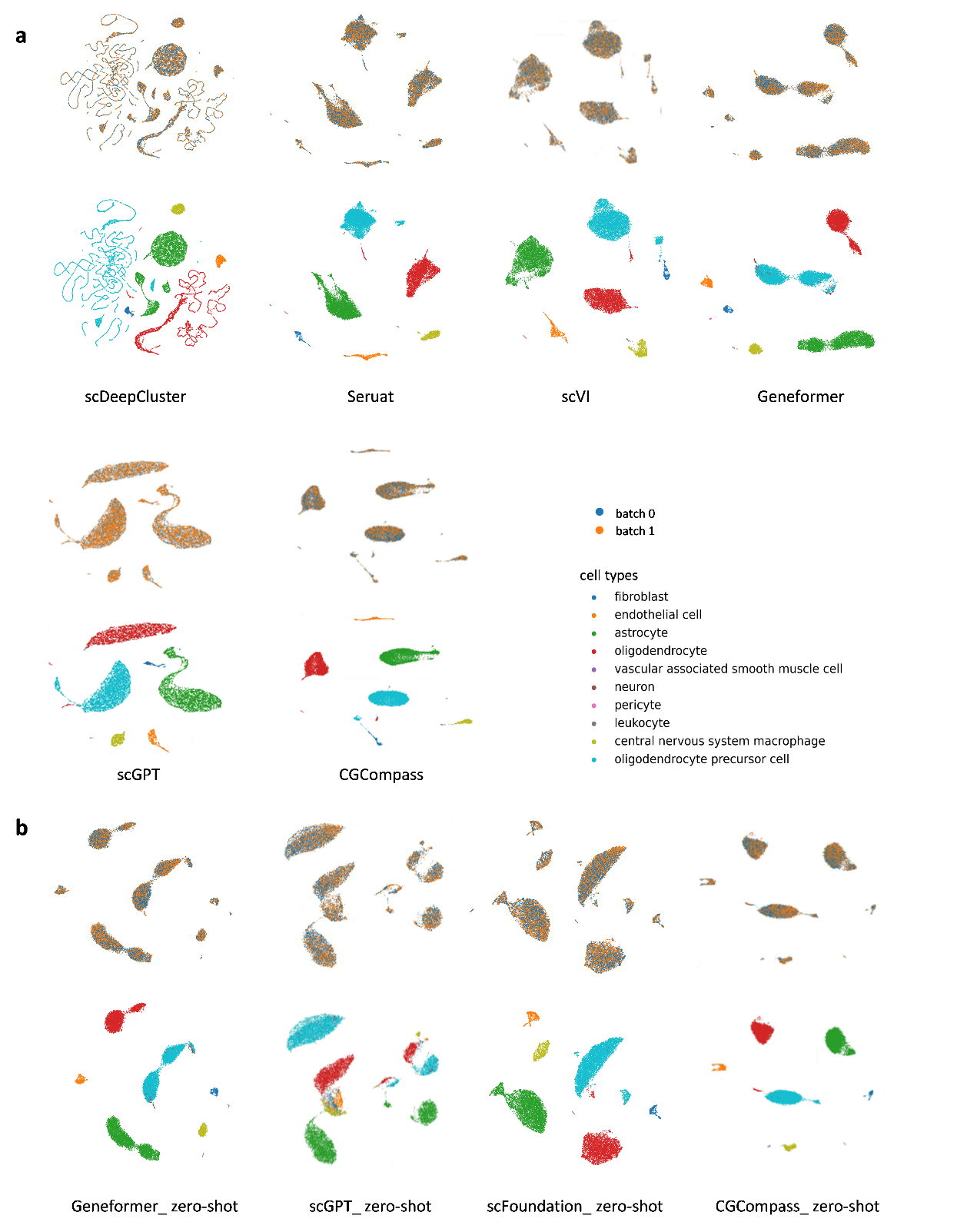


**Fig. S**3: **Batch integration results on the PCortex dataset.** a, UMAP plots of cell embeddings generated by CGCompass and the baseline models. The upper plots are colored by batch labels, and the lower plots are colored by cell types. b, UMAP plots of zero-shot experiments using different foundation models.


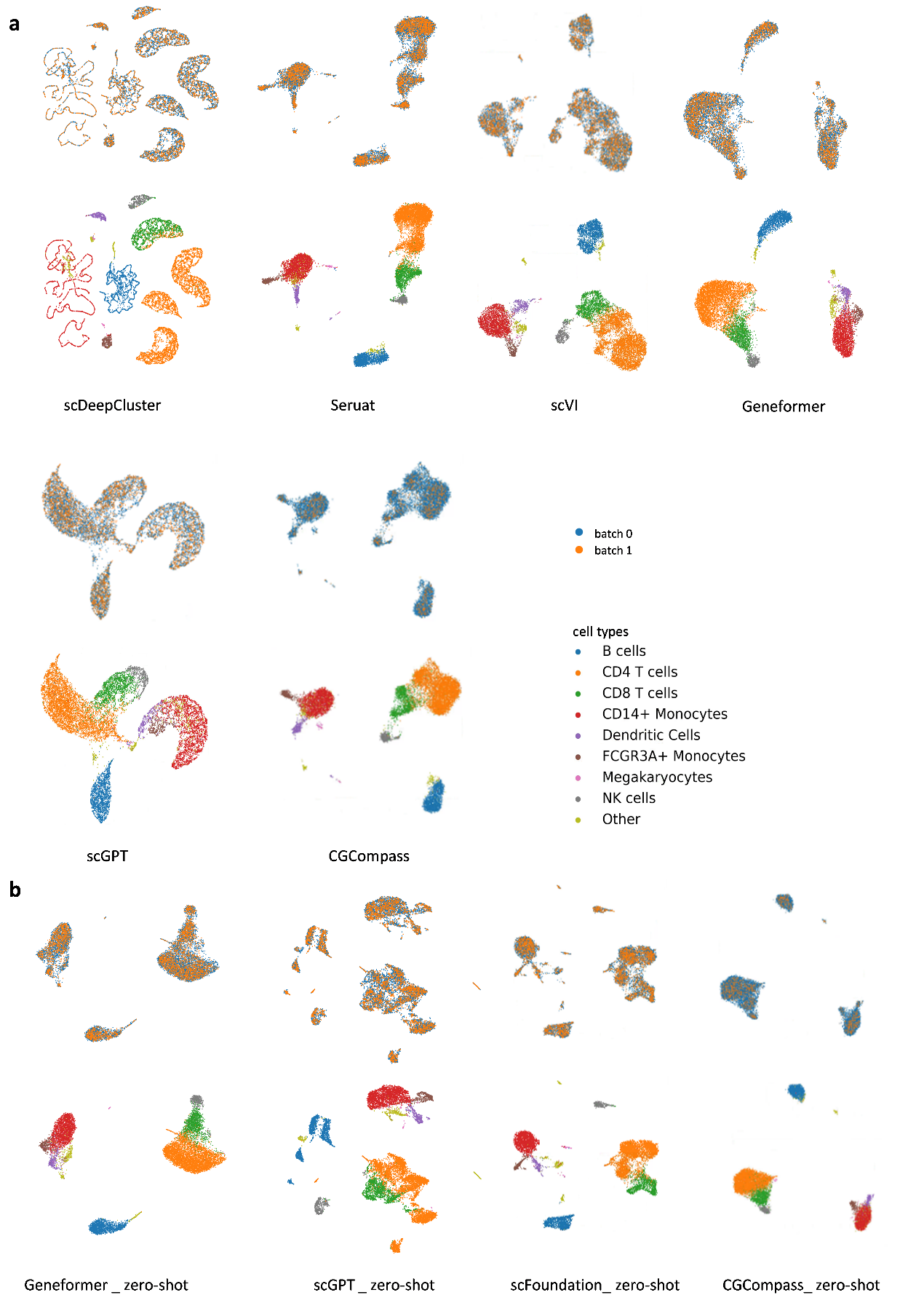


**Fig. S**4: **Batch integration results on the PBMC dataset.** a, UMAP plots of cell embeddings generated by CGCompass and the baseline models. The upper plots are colored by batch labels, and the lower plots are colored by cell types. b, UMAP plots of zero-shot experiments using different foundation models.


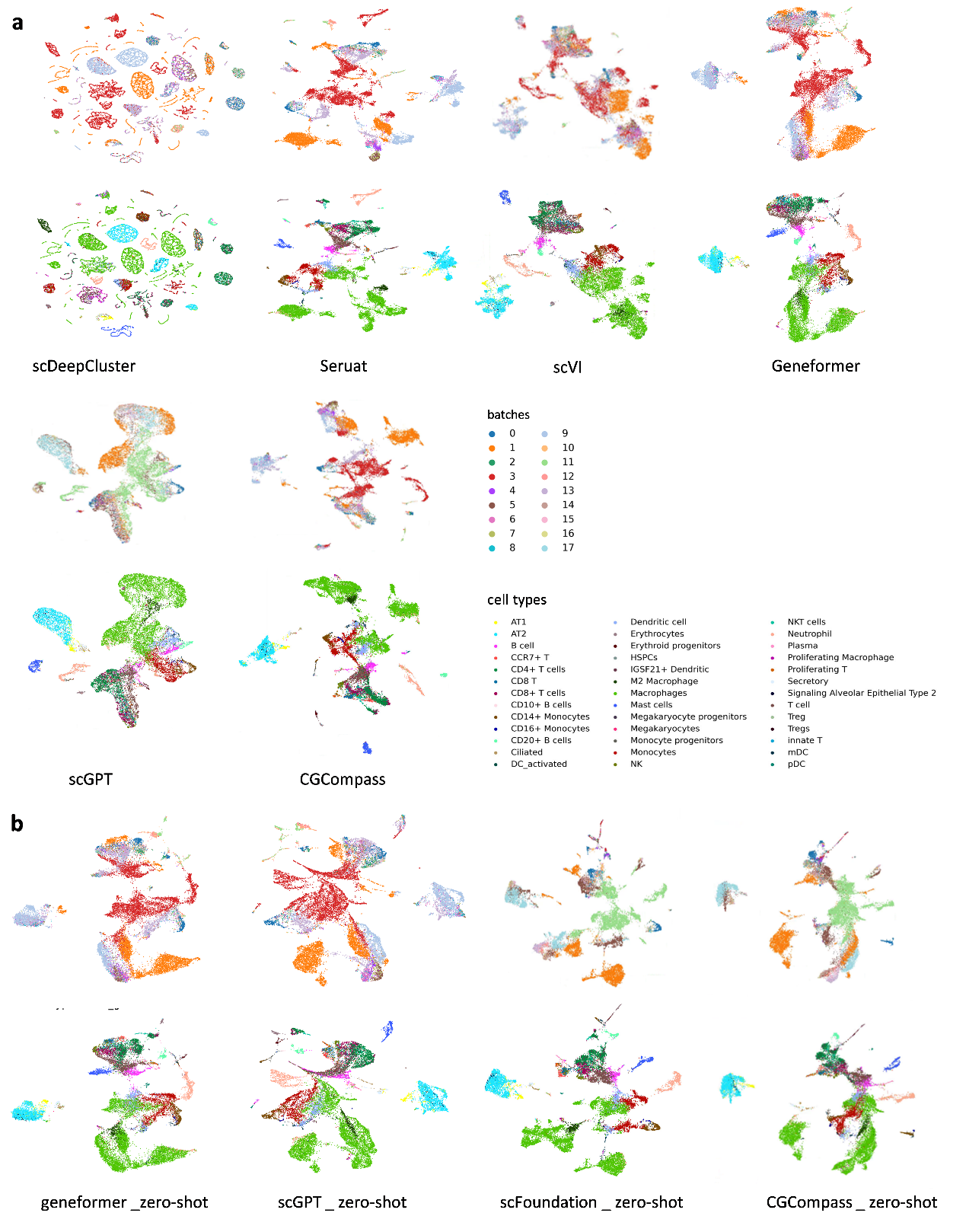


**Fig. S**5: **Batch integration results on the Covid dataset.** a, UMAP plots of cell embeddings generated by CGCompass and the baseline models. The upper plots are colored by batch labels, and the lower plots are colored by cell types. b, UMAP plots of zero-shot experiments using different foundation models.


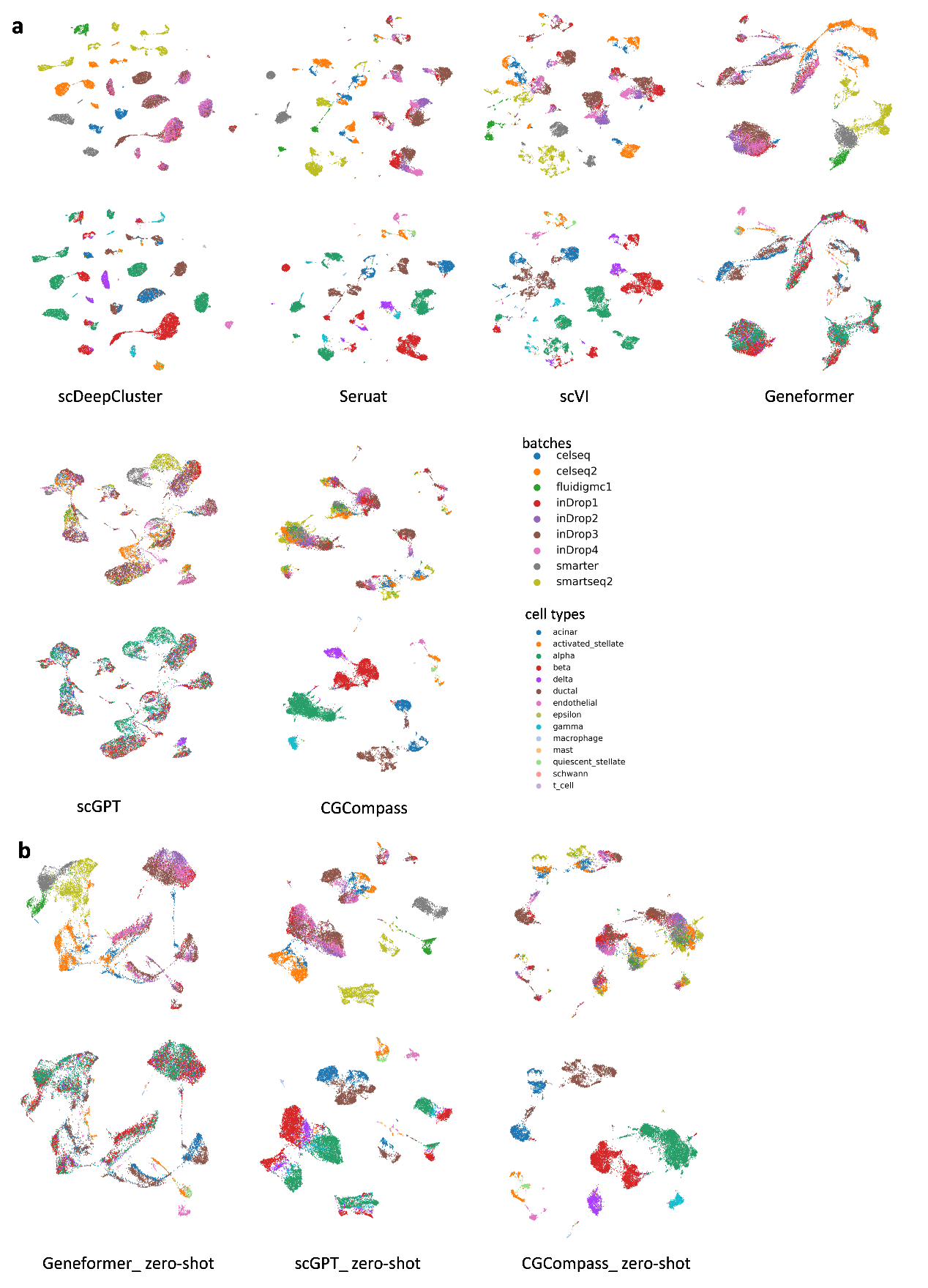


**Fig. S**6: **Batch integration results on the human Pancreas dataset.** a, UMAP plots of cell embeddings generated by CGCompass and the baseline models. The upper plots are colored by batch labels, and the lower plots are colored by cell types. b, UMAP plots of zero-shot experiments using different foundation models.


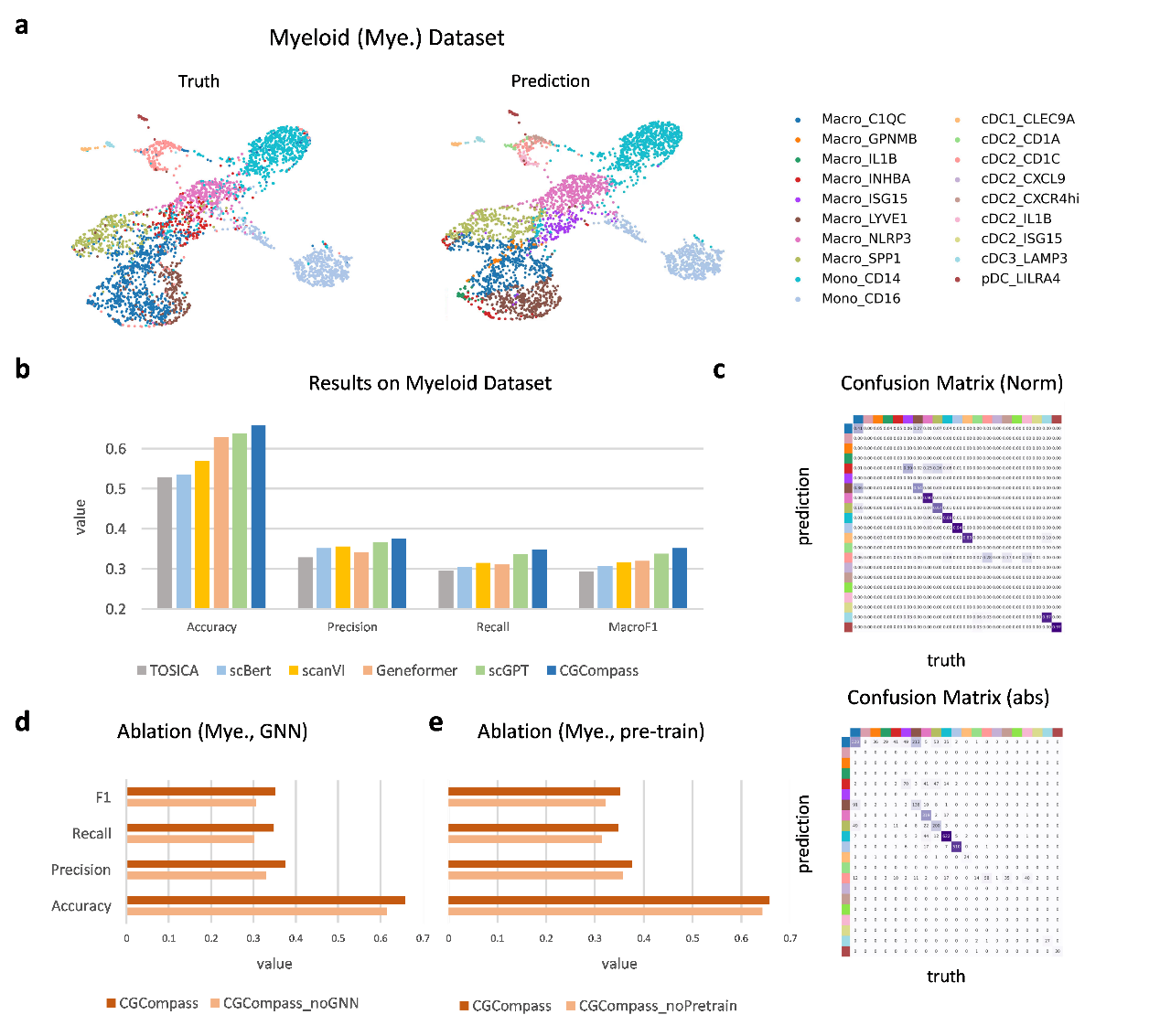


**Fig. S**7: **Results of Cell Type Annotation on the Myeloid (Mye.) dataset.** a, UMAP visualization of cell embeddings generated by CGCompass, colored by ground truth cell types (left) and CGCompass prediction results (right). b, Quantitative evaluation of cell annotation by CGCompass and baseline models. c, Confusion matrix between cell types predicted by CGCompass and ground truth labels, with normalization on the top and absolute values on the bottom. d, Ablation study on the graph structure of CGCompass. e, Ablation study on the pre-training process of CGCompass.

**
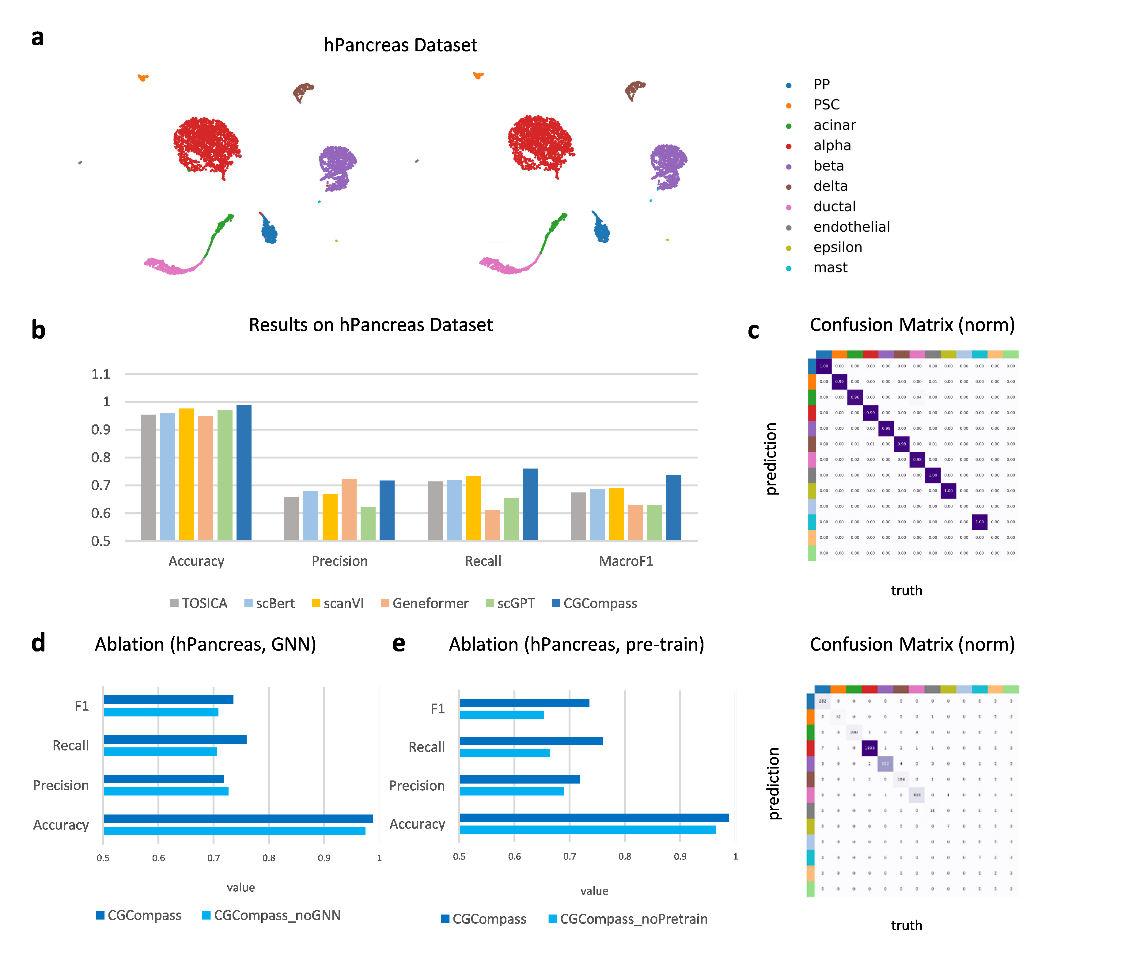
**

**Fig. S**8: **Results of Cell Type Annotation on the hPancreas dataset.** a, UMAP visualization of cell embeddings generated by CGCompass, colored by ground truth cell types (left) and CGCompass prediction results (right). b, Quantitative evaluation of cell annotation by CGCompass and baseline models. c, Confusion matrix between cell types predicted by CGCompass and ground truth labels, with normalization on the top and absolute values on the bottom. d, Ablation study on the graph structure of CGCompass. e, Ablation study on the pre-training process of CGCompass.


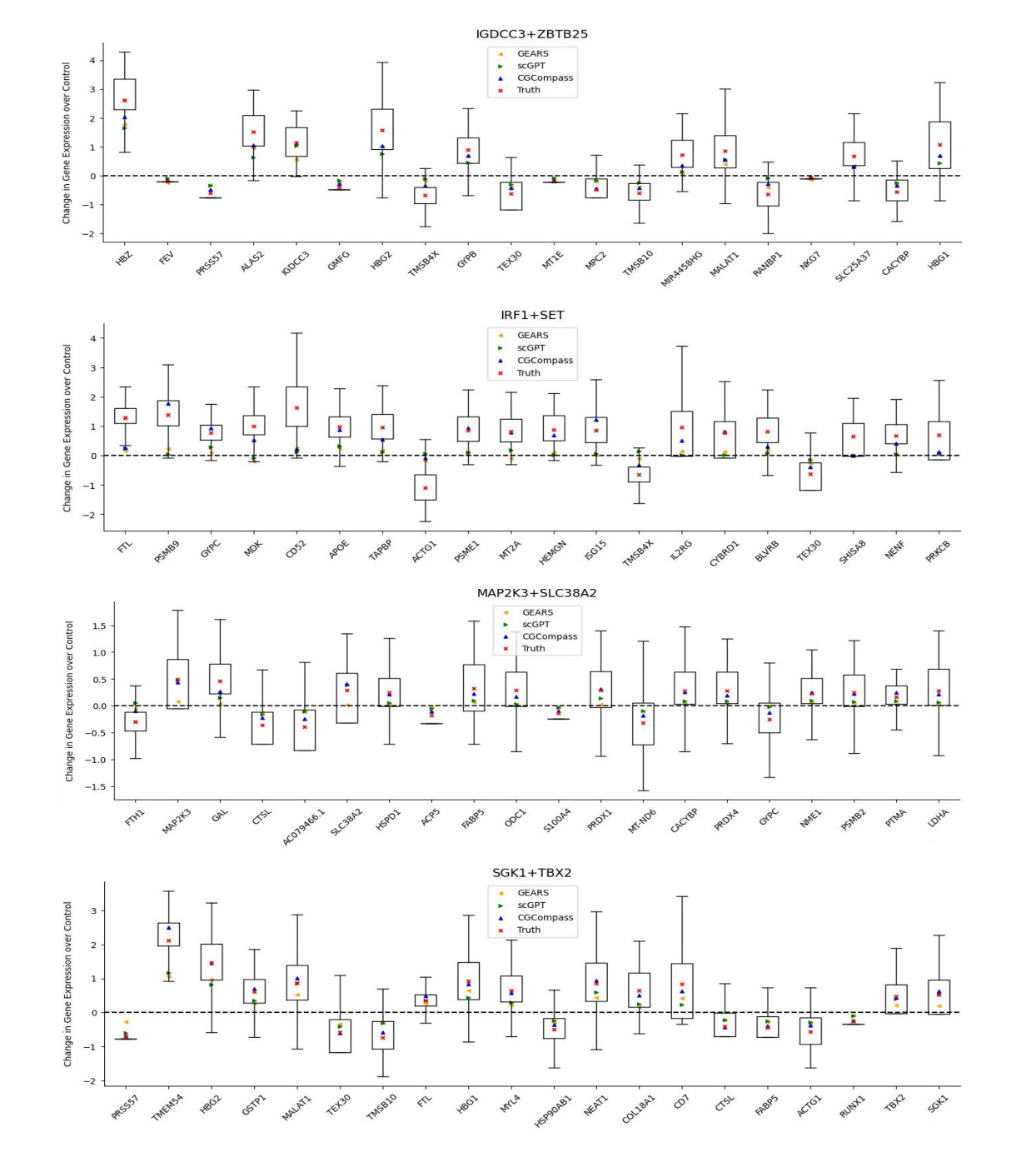


**Fig. S**9: **Examples of gene perturbation response prediction.** From top to bottom, the perturbation conditions are for IGDCC3 and ZBTB25, IRF1 and SET, MAP2K3 and SLC38A2, SGK1 and TBX2, respectively. The x-axis shows the top 20 differentially expressed (DE) genes under each perturbation condition, and the y-axis indicates the change in gene expression due to the perturbation. The box plots represent the distribution of real experimental data for each perturbation condition, with red dots indicating their mean values. Other colored dots represent the prediction values from various models.


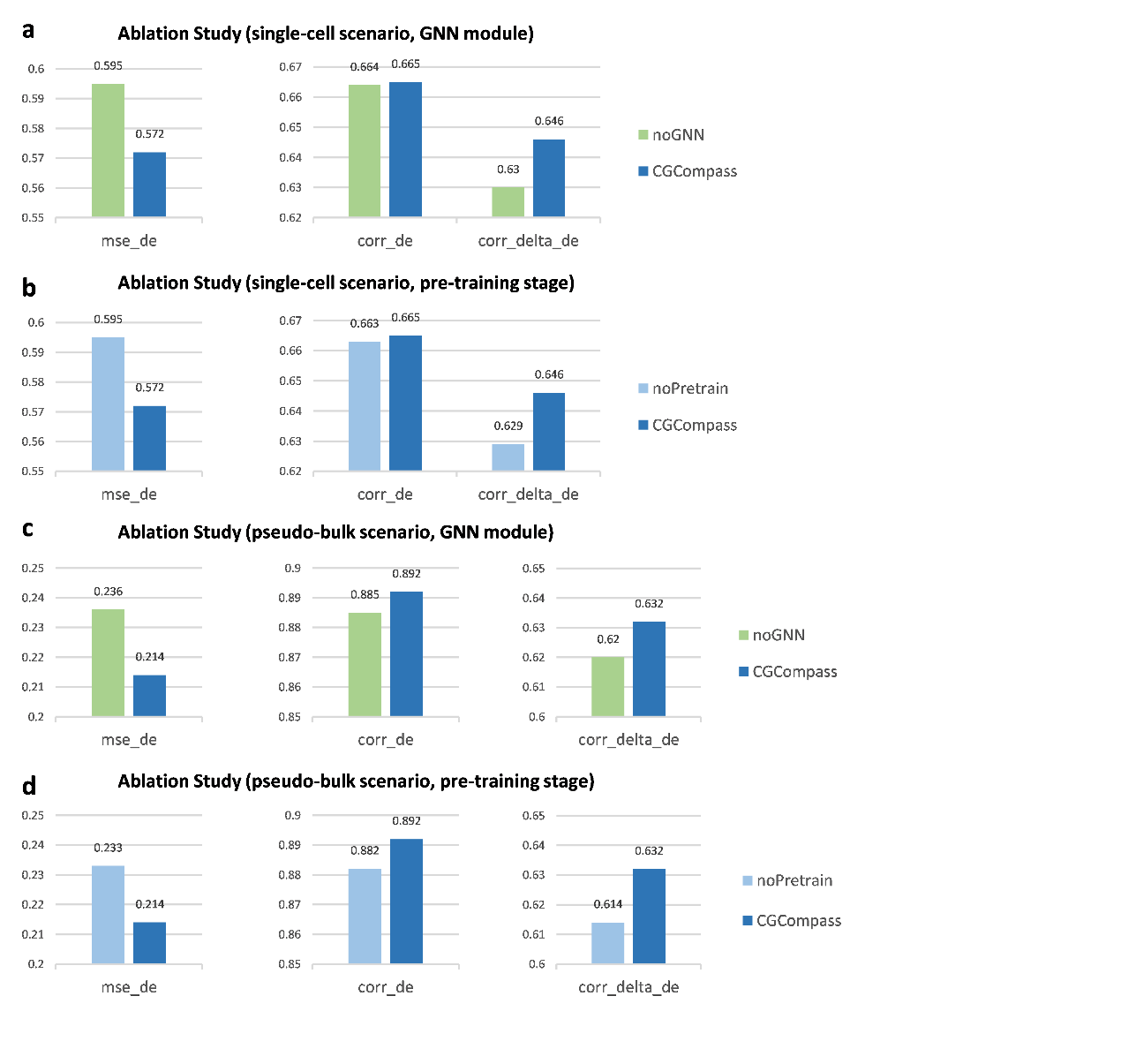


**Fig. S10: Ablation experiments of CGCompass in the single-cell gene perturbation experiment.** On the test set of the Norman dataset: a, Ablation study on the GNN module (single-cell scenario). b, Ablation study on the pre-training process (single-cell scenario). c, Ablation study on the GNN module (pseudo-bulk scenario). d, Ablation study on the pre-training process (pseudo-bulk scenario).

**
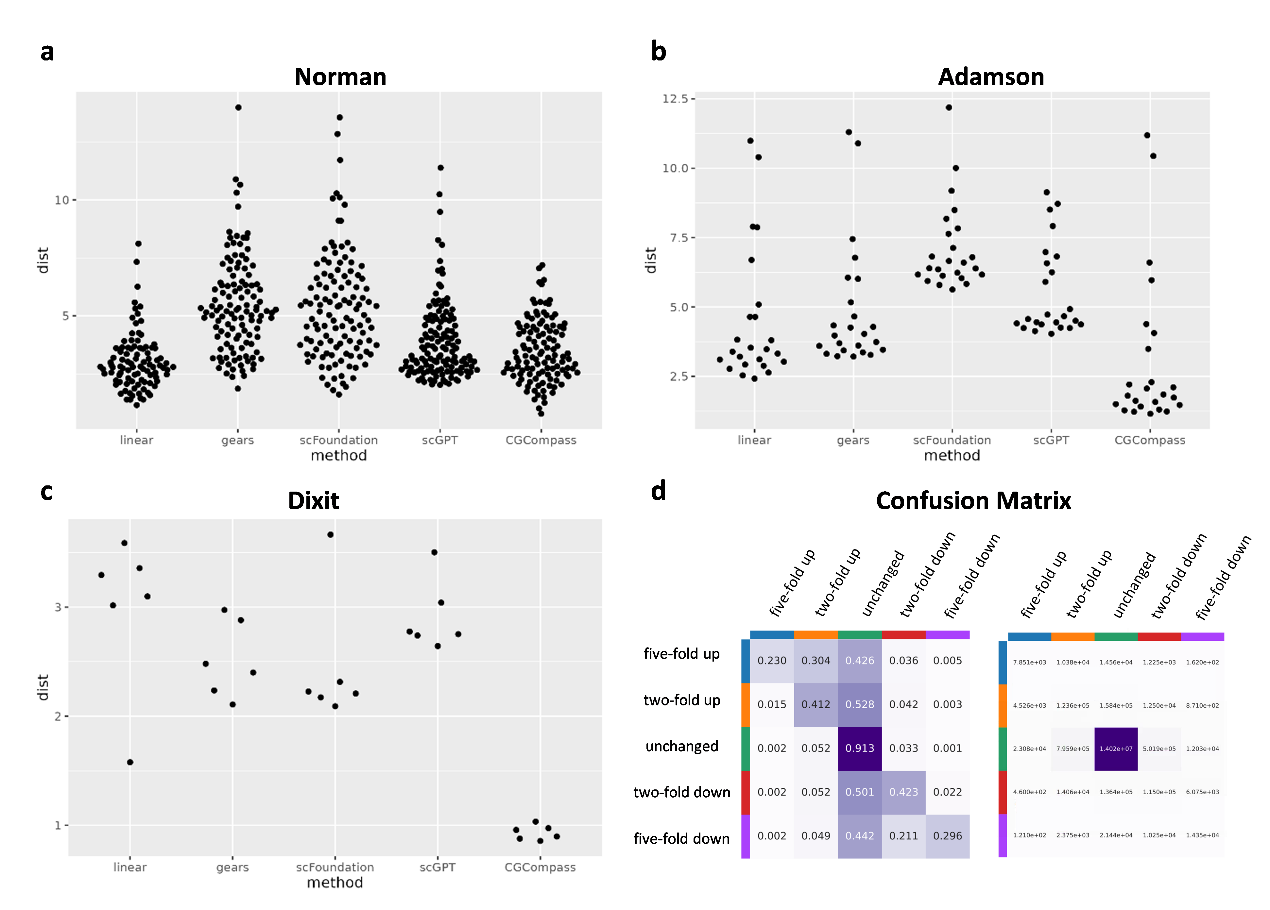
**

**Fig. S11: Additional analysis of perturbation prediction experiments.** **a-c.** Mean squared error (MSE) between predicted and ground truth gene expression across three datasets used for single-cell gene perturbation prediction tasks. Each point represents a distinct perturbation condition within the dataset. **d.** Confusion matrix for the five-class classification task (five-fold upregulation, two-fold upregulation, no change, two-fold downregulation, five-fold downregulation) in the bulk knockout experiments. The left panel shows the normalized proportions, while the right presents the absolute counts.
