# Supplementary material for: Cell-GraphCompass: modeling single cells with graph structure foundation model
Source: Natl Sci Rev. 2025 Jun 24;12(10):nwaf255. doi: 10.1093/nsr/nwaf255 (PMC12485608; doi:10.1093/nsr/nwaf255)
Supplement: nwaf255_Supplemental_Files [file nwaf255_supplemental_files.zip › Supplementary Methods.docx]

# Supplementary Methods

**S1 Constructing cell graphs using biological features**

CGCompass abstracts each cell as a topological graph , where the node set consists of the genes of interest, which may vary across different application scenarios. The edge set contains the relationships between these genes, which are derived from the prior knowledge that we have collected. Let denote node features, anddenote edge features.

**Node features** For each gene  in cell, CGCompass uses three types of biological features describing this gene’s information as its node features ​.

- **Gene tokens  ​:** Each RNA-seq data fed into CGCompass contains a combination of genes. To identify the unique identities of these genes, we have assigned a global ID to each gene encountered during the pre-training process. Analogous to the concept of ‘word tokens’ in NLP, we refer to these gene IDs as 'gene tokens'. The use of gene tokens enhances the flexibility of the model’s input. For new genes, simply assigning a new ID allows their seamless integration into the model’s training framework. For existing genes, the iterative feature learning of gene tokens maintains the model’s 'memory' of these genes, ensuring continuity and precision in gene-related data processing.
- **Gene text descriptions ​**: Currently, there is an abundance of research focused on gene functions accompanied by extensive textual descriptions. Mining these existing textual resources is advantageous for rapidly acquiring some preliminary, foundational information about genes. We have obtained textual descriptions of various genes from the NCBI gene database68, and these descriptions have been transformed into 768-dimensional feature vectors  ​by querying BioBERT15, a specialized language model for the biomedical domain.
- **Gene expression values**: The expression levels of genes within a cell reveal this cell’s type and its current state. Providing the model with information on gene transcription expression is crucial for acquiring features that are relevant to this cell’s context. In downstream applications, CGCompass accepts normalized and log-transformed transcript expression values  as input. During the pre-training stage, given the diverse sources of the pre-training corpus and the significant differences in sequencing depth and gene capture rates, we additionally use *value binning* to standardize the data scale. We arrange all non-zero expressed genes in a cell according to their expression and bin them into consecutive intervals , where . Each interval contains an equal number of genes. The binning value for gene  is then given by: .
- **Special tokens**: This feature is designed to facilitate the embedding of additional information in specific scenarios. For instance, in the context of perturbation prediction, we use a '0/1' indicator to denote whether a gene has been perturbed or not. In this paper, we employ this feature exclusively in two downstream tasks: single-cell gene perturbation prediction and bulk knockout prediction.

Therefore, gene 's input node features can be represented as (, ,).

**Edges features.** Edge in cell graph represents relationships between gene and gene . CGCompass collected three types of biological features to depict these gene relationships.

- **Transcription Factor (TF)-Target Gene (TG) interactions** :Research on TFs and their target genes has been conducted for many years.Embedding the results of previous research as prior knowledge into our model is expected to significantly enhance the performance of CGCompass. We have obtained pre-existing TF-TG relationships from two widely recognized databases: TFBSDB database1 and TRRUST2. The former contains TF-TG regulatory relationships inferred from experiments, while the latter summarizes regulatory relationships documented in the literature. We generate directed edges for the genes within each cell, which start from transcription factors and point to their target genes.
- **Gene co-expression relationships** : The transcriptional activation patterns of genes help reflect their functions or characteristics. If the expression patterns of two genes are particularly similar, they may have similar functions or interactions with each other. We employ the Pearson Correlation Coefficient (*corr*) as a measure of gene co-expression relationships and retain gene pairs with a *corr* value greater than 0.6 to form the second type of edges. During the pre-training stage, the *corr* is calculated separately for each SRR experiment, whereas in the downstream task, it is computed across the entire dataset.
- **Positional relationship on chromosomes** : Genes located in closer proximity on the same chromosome tend to exhibit higher similarity. We have calculated the relative distances between all gene pairs on the chromosomes (measured in terms of the number of genes separating them), and retained those gene pairs with a relative distance of less than 50 to form the third kind of edges.

Therefore, the input features of each edge can be marked as (, , ).

**S2 CGCompass model architecture**

**Embedding module.** For the different types of feature data inputted into CGCompass, we have designed specific encoders to integrate all information into unified feature vectors for nodes and edges. Gene tokens, special tokens, TF-TG interactions, and positional relationship on chromosomes are encoded using traditional embedding layers： , , , , while gene text descriptions, gene expression values, and gene co-expression relationships are encoded using three-layers Multi-Layer Perceptions(MLPs): , , . These encoded features are then combined using an element-wise sum with equal weights (1:1:1)：

**GNN module.** Adhering to the message passing paradigm, we have developed a three-layer GNN module to jointly learn the feature representations of nodes and edges. In each layer, the information of adjacent nodes and connecting edges is utilized to update the central node. Subsequently, the central edge’s information is updated using the data from the edge itself and its connected nodes. The process is articulated through the following formulas:

.

where and represent the latent vectors of nodes and edges output by the -th layer, with = and = denote the input of the GNN module. refers to the set of neighboring nodes of gene . and both refer to a three-layer MLP while MEAN(·) denotes the operation of calculating the average.

**Transformer module** Next, we designed a self-attention Transformer module to capture the interactions between genes from a global perspective. Specifically, we first derive the query, key, and value matrices from the node features outputted by the GNN module:

**.**

where are learnable parameters, with and represent the dimension of latent vectors. Then, we apply self-attention mechanism among all nodes:

here, represents the feature representation outputted by the transformer module for each gene. In addition to the traditional transformer model architecture, we have also implemented *Flash-Attention*3 technology to accelerate the attention mechanism.

**Cell embedding.** Once we have obtained the embeddings for all the genes within a sequencing sample, we can synthesize these to form an overall representation of this cell. This could be achieved by simply averaging or performing a weighted sum of all genes’ embeddings. However, in this paper, we adopted a more sophisticated approach. We introduced an additional node to the graph representing the overall state of the cell, with all other nodes representing genes connected to it. This allows both the GNN and the Transformer module to autonomously learn the integrated representation from the gene representations. The feature representation outputted by the Transformer for this node is then extracted as the cell embedding.

**S3 The pre-training strategy of CGCompass**

Weemployed a traditional masked training strategy for self-supervised learning on scCompass-h50M. For each input RNA-seq data, we randomly mask 40% gene expression values and predict these masked values using the expression of the remaining genes. This process can be formalized as follows:

where and represent the actual and predicted expression values of gene in each cell, and indicates the gene embedding output by the Transformer module. is the set of indices for the masked genes, and denotes the mean squared error operation.

For the learning of cell embedding, we attempted to predict the expression values of all genes using cell embedding and gene tokens . The process is formalized as follows:

where represents the prediction by cell embedding and ⟨·,·⟩ signifies inner product operation.

**S4 Downstream tasks**

**Gene classification.** To explore whether CGCompass’s pre-training can learn meaningful biological knowledge, we extracted the gene token embeddings from pre-trained CGCompass for each gene. We attempted to use these embeddings to distinguish the identity or characteristics of certain specific genes, using six gene classification datasets compiled by Theodoris et al7:

- **Dosage sensitive prediction** tests whether a gene is sensitive to its dosage, which is essential for understanding the mechanisms of genetic diseases caused by gene dosage imbalances. In our experiments, we used previously reported17-19 481 genes for experiments, including 121 positive samples and 358 negative samples**.**
- **Transcription factors’ action range** reflects different regulatory modes by the distance of TF binding to target DNA sequences. We utilized a dataset from Chen et al.21, comprising 173 samples, including 46 positive samples and 127 negative samples.
- **Bivalent chromatin structure** involves a unique epigenetic modification at some gene promoters, significant in gene expression regulation, especially during early development and stem cell differentiation. The bivalent domain combines two opposing histone modifications: H3K4me3 and H3K27me3. Using data on 56 conserved regions from previous reports20, we first differentiated between bivalent and Lys4-only methylated genes, then further between bivalent and non-methylated genes, with dataset sizes of 147 (40 positive and 106 negative) and 187 (106 positive and 78 negative), respectively.
- **Network dynamics prediction** involves identifying core genes within gene regulatory networks, a key to understanding the mechanisms of certain diseases. We utilized a NOTCH1 (N1)-dependent gene network provided by Theodoris et al.22,23, aiming to predict which genes are core to the N1 network and which are peripheral downstream effectors. Furthermore, we sought to predict which genes are influenced by this N1 network. The sizes of the two datasets are 281 (97 positive and 183 negative) and 1103 (557 positive and 554 negative), respectively.

We generated embeddings for each gene using Gene2vec, BioBERT and CGCompass, along with random embeddings for comparative experiments. For each method’s embedding, we uniformly employed a Logistic Regression classifier, evaluating models’ performance through five-fold cross-validation. We selected four evaluation metrics to assess the model performance: F1-score, ROC-AUC, AUC_PR, and the Kappa coefficient.

- **F1-score** are macro-averaged, meaning scores for positive and negative samples are calculated separately and then averaged to obtain the final result.
- **ROC-AUC** (Area Under the Curve) calculates the area under the ROC curve. The ROC (Receiver Operating Characteristic) curve plots the True Positive Rate (TPR) on the vertical axis and the False Positive Rate (FPR) on the horizontal axis. ROC-AUC ranges from 0 to 1, where 1 indicates perfect prediction, 0.5 indicates random prediction, and 0 represents a completely worthless prediction.
- **AUC_PR** computes the area under the Precision-Recall (PR) curve. The PR curve has Precision on the vertical axis and Recall on the horizontal axis. Since both Precision and Recall measure the model's ability to identify Positive Samples, AUC_PR is more suitable when focusing on Positive Samples. AUC_PR also ranges from 0 to 1, with higher values indicating better performance.
- **Kappa** **coefficient** is calculated as ​​, where ​ is the proportion of samples correctly classified across all categories (overall classification accuracy), and is the sum of the product of the “actual quantity” and “predicted quantity” for each class, divided by the square of the total number of samples. The Kappa coefficient ranges from -1 to 1, though it typically falls between 0 and 1. A higher Kappa value indicates greater agreement between the predictions and the true labels.

**Gene-Gene Interaction prediction.** This task aims to determine whether there is an interaction relationship between two genes. Our data is derived from two databases, STRING25 and ChIP-seq26-28, covering two cell types: human embryonic stem cells (hESC) and human mature hepatocytes (hHEP). Following work by Pratapa et al.4, we conduct experiments with the top 500 and 1,000 genes that exhibit the highest variance among all TFs, with a corrected P-value for variance below 0.01. Similar to the inference method of DeepSEM29, we use the cosine similarity between gene pairs as the basis for prediction:

where ⟨·, ·⟩ signifies the inner product operation and ∥·∥ represents the calculation of the Euclidean norm. We evaluate the model’s ability to predict the interaction between these genes. First, we retain the top gene pairs with the highest cosine similarity, where represents the number of edges in the ground-truth interaction network. We then calculate the proportion of our retained gene pairs that overlap with the ground-truth network, as depicted by the Early Precision Ratio (EPR) metric. Secondly, based on the similarity of gene pairs, we have the model output the probability that there is a interaction relationship between them, quantified by the Area Under the Precision–Recall Curve (AUC_PR) ratio, to measure the model’s capability to predict edges present in the ground-truth GRN.

**Gene programs.** The extraction of gene functional clusters was conducted on the human immune tissue dataset. Our validation steps follow the approach of Cui et al.8: Firstly, we selected highly variable genes and constructed a gene similarity network based on the similarity between gene pairs, as with Equation 7. Subsequently, we applied Louvain clustering5 to the resulted network at a resolution of 40 in order to divide these genes into different gene programs. Finally, we calculated the average expression of each program across various cell types.

**Batch integration.** This task aims to differentiate between data from various cell types across different sequencing batches. The downstream datasets have undergone normalization, log-transformation, and highly variable gene extraction (1200 genes). Notably, we did not perform value binning in this case, as the datasets are less complex than the pre-training corpus and using absolute gene expression values may preserve more complete information.

In the zero-shot experiment, the model is directly tested, whereas the fine-tuning experiment employs the same masking training method as in the pre-training phase. In addition to the two losses from pre-training (Equation 5 and 6), the overall loss function for fine-tuning CGCompass includes two additional losses to aid batch integration. Domain Adaptation via Reverse Back-Propagation employs an MLP classifier to predict the batch to which a cell belongs, based on the cell embeddings output by the model. The gradient of this prediction loss is reversed during backpropagation, aiming to reduce the influence of batch effects on the cell embeddings. Elastic Cell Similarity aims to make similar genes more similar and dissimilar genes more distinct. These two methods were proposed and used by scGPT.

For comparison in the fine-tuning experiment, we selected scDeepCluster34, Seurat35, scVI33, Geneformer, and scGPT as baseline models. The evaluation metrics we used are the biological conservation and batch effect correction metrics proposed by Luecken et al.30:

- **Normalized Mutual Information (NMI)**: NMI is used to measure the amount of shared information between clustering results and cell type labels. Through normalization, the value of NMI is constrained between 0 and 1, where 0 indicates that the two datasets are completely independent, and 1 indicates that the datasets are completely related.
- **Adjusted Rand Index (ARI)**: The Rand Index (RI) is an indicator used to measure the consistency between clustering results and groud-truth cell type labels by comparing the consistency and inconsistency of paired combinations in the clustering allocation. ARI is an improved version of RI, adjusted to account for the effects of random classification. The value of ARI ranges from 0 to 1, where 1 represents a perfect clustering effect, and 0 indicates the result of random clustering.
- **Average Silhouette Width (ASW)**: The silhouette coefficient is used to calculate the difference between the average distance of a sample to other samples in the same category (cohesion) and the average distance to samples in the nearest other category (separation). We use batches as category labels and take the complement of the generally defined ASW as our indicator for depicting batch confusion: . Under this definition, the ASW ranges from 0 to 1, with higher values representing better batch effect correction.
- **Graph Connectivity (GraphConn)**: This metric measures the average proportion of samples within each cell type that are connected through the K-Nearest Neighbors method. The value of GraphConn ranges from 0 to 1, representing the model’s ability to overcome batch effects.

The average of these four metrics is considered the overall score.

The Perirhinal Cortex (P.C.) dataset includes sequencing data from ten cell types across two batches32. The PBMC 10K dataset 36 contains data from two sequencing batches of human peripheral blood mononuclear cells, covering nine cell types. The COVID-19 dataset, derived from recent studies37, includes data from 18 batches and 39 cell types. The human pancreas dataset comes from nine different sequencing platforms and covers 14 cell types30.

**Cell type annotation.** Cell type annotation is a classification task at the cellular level. Our objective is to predict each RNA-seq sample’s cell type based on its gene expression profile. CGCompass employs a three-layer MLP as the decoder for this scenario, which uses the cell embeddings as input to generate predictions. This process can be mathematically represented as follows:

where and respectively represent the actual and predicted values of this RNA-seq sample. represents the operation of calculating cross-entropy. We employ traditional classification metrics such as *accuracy*, *precision*, *recall*, and *F1 score* to quantitatively evaluate the performance of tested models. Notably, the *accuracy* metric is calculated globally (i.e., *micro*), while the other metrics represent the average outcomes across different cell types (i.e., *macro*).

**Single-cell gene perturbation prediction.** This task can be defined as follows: given a set of gene expression profiles from cells under control (unperturbed) conditions and their respective perturbation conditions (specifying which genes will be perturbed), predict the corresponding gene expression profiles post-perturbation. In this application scenario, we employ the special tokens mentioned in Method 4.1 and adopt the complete transcript expression values as input. CGCompass outputs an embedding representation for each gene and uses a shared decoder across these representations to determine their post-perturbation expression levels. During the fine-tuning process, the loss function for each perturbation sample can be defined as:

where denotes the shared decoder and represents the ground-truth value.

The gene-wise correlation test calculates the *corr* between the predicted and actual expression profiles for each gene across all cells. We then compared the number of genes for which CGCompass outperformed GEARS, as well as the number of genes for which CGCompass outperformed scGPT.

Furthermore, we calculated the accuracy of whether the average predicted values for DE genes fall within the central region of the true value distribution (Fig. 5g). Specifically, for each perturbation condition, we treat the average predicted gene expression as a point and consider the true values as a distribution. We then calculate the accuracy of whether the prediction points for all DE genes across all perturbations fall within the central region of this distribution. The distribution center is defined as the interval between the values at the and percentiles after ordering all true values.

**Bulk-level gene knockout prediction.** The bulk-level gene knockout is a downstream task that, while similar in definition to single-cell gene perturbation, holds an entirely different significance. In single-cell perturbation experiments, our focus is often limited to samples under specific cell conditions, with an emphasis on enhancing prediction accuracy. However, in bulk knockout prediction experiments, our aim is to develop a more universal predictive model applicable to a broader range of cell conditions, in hopes of uncovering universal relationships between genes.

To transfer the single-cell pre-trained model to the bulk knockout scenario, we employed a strategy of secondary pre-training. We downloaded 700k high-throughput bulk sequencing data entries of mice from the ARCHS46 database, retaining 300k after quality filtering to serve as our bulk-level pre-training dataset. These sequencing data are organized in FPKM format and have undergone log1p transformation and sampling based on expression values, among other processes. The final input for the model consists of 2048 gene tokens per entry.

In building the bulk knockout dataset, we initially extracted sequencing results related to knockout experiments from NCBI’s GEO16 database. We then categorized these sequencing results individually based on the experimental condition according to overall design of these GSE experiments and the different treatments of GEM samples. Ultimately, we retained the average expression of all samples under each experimental condition as a single entry in our bulk knockout dataset. Hence, some entries in our dataset, despite having the same knocked-out gene, exhibit significant data distribution differences due to their varying experimental conditions. The training set comprises 2,368 single-gene knockouts, 235 double-gene knockouts, and 39 multi-gene knockouts. The test set includes 512 single-gene knockouts, 42 double-gene knockouts, and 7 multi-gene knockouts. Notably, there is no overlap in experimental conditions between the training and test sets, ensuring robust evaluation.

We first retrained our single-cell pre-trained model on the 300k bulk sequencing data using the same masked training loss mentioned in the Formula 5 and 6, and then fine-tuned it on the bulk knockout dataset using cross-entropy loss function. To address the issue of too few up-/down-regulated genes in the ground-truth data, we adopted a “secondary prediction” strategy. For each gene, we first predict whether it will change, and then for each gene that changes, we predict the direction of its change. This process can be expressed in the following formula:

where denotes the set of changed genes after knockout. is the weight coefficient that coordinates the proportion between and . indicating whether the gene changes, and specifying the direction of the change. FOCAL(·) represents focal loss, which is designed to address class imbalance by reducing the loss contribution from easy-to-classify samples, thereby focusing more on hard-to-classify samples. We used the scGPT encoder connected to our two-step classification decoder as a baseline. Additionally, we employed a Transformer encoder with 12 layers, 8 attention heads, and a 512-dimensional space, connected to our decoder as another baseline.

**S5 Implementation Details**

The GNN module of CGCompass is stacked with 3 layers, and the Transformer module is stacked with 12 blocks with 8 attention heads each. The embedding size and the fully connected layer hidden size are both 512. We use scCompass-h50M dataset for pre-training, where 99% of the data is used for training and 1% for validation. We use the mini-batch size of 32 with the gradient accumulation step number for 2, and a total of 8 A100-40G GPUs were used for 3 epochs of training. Note that only non-zero expression genes are input in the pre-training stage, and the max sequence length was set to 1200. The mask ratio is set to 0.4 and the ratio of (Formula 5) to (Formula 6) is set to 1. The model uses the Adam optimizer with Adam betas set to (0.9, 0.999) and eps of 1e-8. The model used warmup and exponential decay to adjust the learning rate. The max learning rate is set to 1e-4, the warmup steps is 5000, the decay steps is 1000, and the decay ratio is 0.995.

For the fine-tuning downstream tasks, we keep the same model configuration with the pre-trained model. The cell clustering task uses the batch size of 32 with gradient-free accumulation, and the max sequence length of 1200. The ratio of the training set to validation set is 9:1, and the the ecs threshold of 0.8. For the cell type annotation task, the batch size is set to 8 with the gradient accumulation step number for 2 and the max sequence length is set to 3000. The training set and the validation set were divided by 9:1 on the reference dataset, and 30 epochs of training were conducted using 2 gpus. For single-cell gene perturbation prediction task, the batch size is set for 64 with gradient-free accumulation, and 30 epochs of training were conducted using 1 gpu. As for the bulk-level gene knockout prediction task, the focal loss weight is set to 1, focal alpha is set to 0.5, and other parameters are the same as those of single-cell gene perturbation prediction task.

Both pre-training and fine-tuning experiments used the machine with 8 A100 40G GPUs, 380G of machine memory, and 16 CPU cores. The pre-training efficiency of CGCompass on scCompass-h5M is recorded in Table S46. The *scanpy* and *anndata* package are applied to pre-process single-cell data and we use the *scanpy.pp.highly_variable_genes()* function to extract highly variable genes, with the “*seurat_v3*” flavor selected. The *lmdb* package is used to write pre-processed data into memory for accelerating data reading. We use *pyg* package to implement message passing mechanism and graph data processing, and *flash attention* to avoid length limitation of Transformer. The metrics of cell clustering are calculated and shown by *scib.metrics*, and the metrics of cell type annotation tasks are calculated by *scikit-learn* and shown by *seaborn*. The analysis of single-cell gene perturbation prediction and bulk-level gene knockout prediction is implemented by the *cell*-GEARS package.

**References**

1 Plaisier, C. L. *et al.* Causal Mechanistic Regulatory Network for Glioblastoma Deciphered Using Systems Genetics Network Analysis. *Cell Syst* **3**, 172-186 (2016). https://doi.org/10.1016/j.cels.2016.06.006

2 Han, H. *et al.* TRRUST v2: an expanded reference database of human and mouse transcriptional regulatory interactions. *Nucleic Acids Res* **46**, D380-d386 (2018). https://doi.org/10.1093/nar/gkx1013

3 Dao, T., Fu, D. Y., Ermon, S., Rudra, A. & R'e, C. FlashAttention: Fast and Memory-Efficient Exact Attention with IO-Awareness. *ArXiv* **abs/2205.14135** (2022).

4 Pratapa, A., Jalihal, A. P., Law, J. N., Bharadwaj, A. & Murali, T. M. Benchmarking algorithms for gene regulatory network inference from single-cell transcriptomic data. *Nature Methods* **17**, 147-154 (2020). https://doi.org/10.1038/s41592-019-0690-6

5 Traag, V. A., Waltman, L. & van Eck, N. J. From Louvain to Leiden: guaranteeing well-connected communities. *Scientific Reports* **9**, 5233 (2019). https://doi.org/10.1038/s41598-019-41695-z

6 Lachmann, A. *et al.* Massive mining of publicly available RNA-seq data from human and mouse. *Nature Communications* **9**, 1366 (2018). https://doi.org/10.1038/s41467-018-03751-6
