# Supplementary material for: Cell-GraphCompass: modeling single cells with graph structure foundation model
Source: Natl Sci Rev. 2025 Jun 24;12(10):nwaf255. doi: 10.1093/nsr/nwaf255 (PMC12485608; doi:10.1093/nsr/nwaf255)
Supplement: nwaf255_Supplemental_Files [file nwaf255_supplemental_files.zip › Supplementary Table.docx]

# Supplementary Tables

**S.1 Benchmarking results on downstream tasks**

**S.1.1 Gene Classification**

We selected six binary classification datasets that describe gene characteristics or functions for experimentation. These datasets have relatively small sample sizes and imbalanced distributions, so we performed five-fold cross-validation. The values in the six tables below represent the average of the metrics from the five experiments, with the standard deviations of the five runs shown in parentheses. The top-left corner of each table indicates the abbreviation of the corresponding dataset.


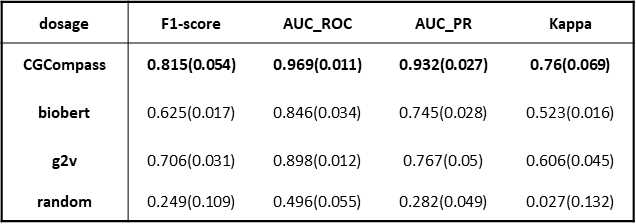


Table S1: Classification results on the dosage sensitive prediction dataset. This dataset contains 121 positive samples and 358 negative samples.


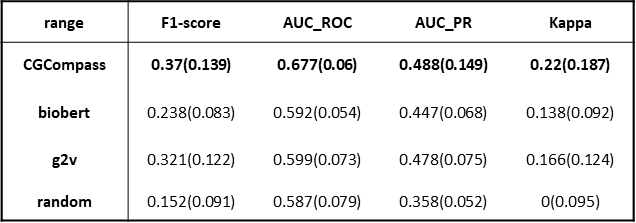


Table S2: Classification results on the Transcription Factors’ action range prediction dataset. This dataset contains 46 positive samples and 127 negative samples.


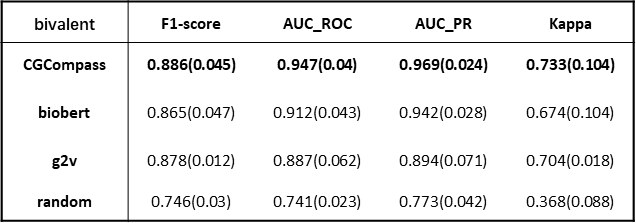


Table S3: Classification results on the bivalent and Lys4-only methylated genes dataset. This dataset contains 40 positive samples and 106 negative samples.


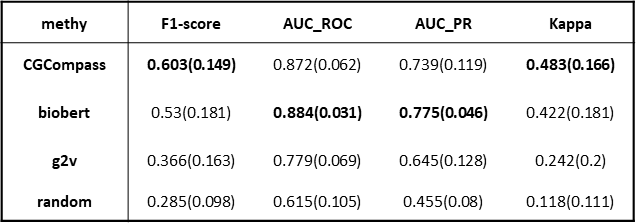


Table S4: Classification results on the bivalent and non-methylated genes dataset. This dataset contains 106 positive samples and 78 negative samples.


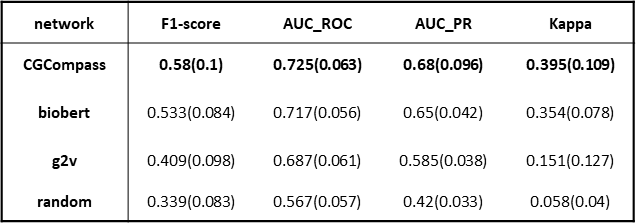


Table S5: Classification results for core genes in the NOTCH1 (N1)-dependent gene network. This dataset contains 97 positive samples and 183 negative samples.


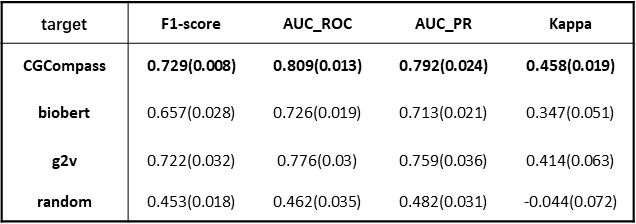


Table S6: Classification results for downstream target genes in the NOTCH1 (N1)-dependent gene network. This dataset contains 557 positive samples and 554 negative samples.

**S.1.1 Batch integration**

We used four evaluation metrics, with the first two assessing the effectiveness of cell type clustering and the latter two evaluating batch mixing performance. The average of these four metrics was used as the final score.


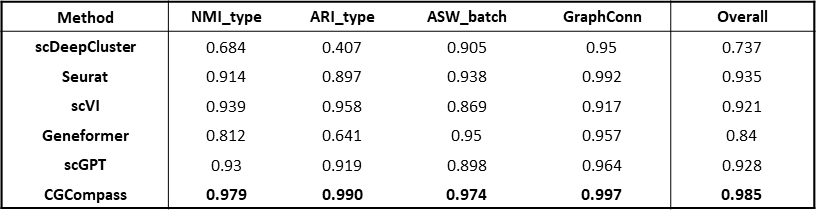


Table S7: Fine-tuning results of batch integration on the PCortex dataset.


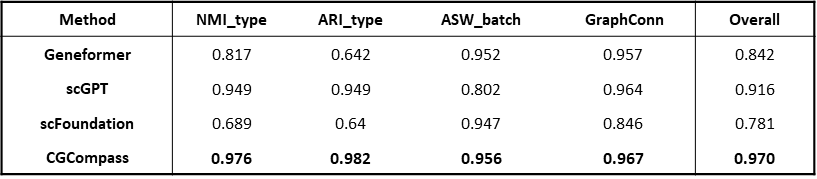


Table S8: Zero-shot results of batch integration on the PCortex dataset.


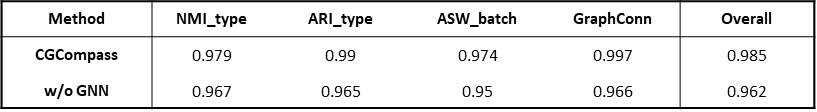


Table S9: Ablation experiments of the GNN module on the PCortex dataset.


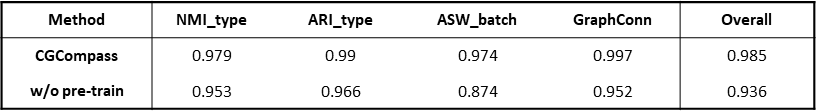


Table S10: Ablation experiments of the pre-training stage on the PCortex dataset.


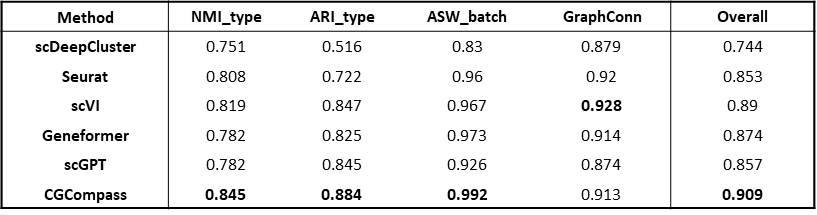


Table S11: Fine-tuning results of batch integration on the PBMC dataset.


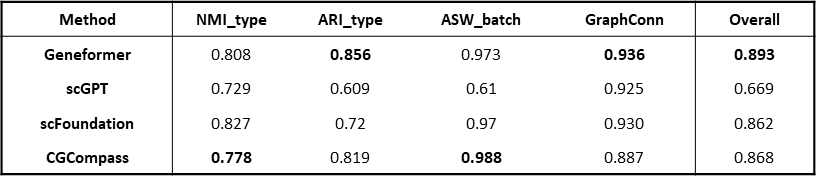


Table S12: Zero-shot results of batch integration on the PBMC dataset.


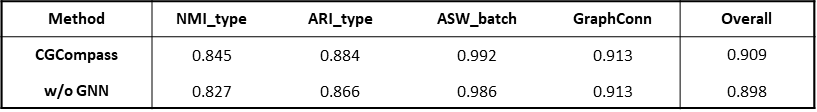


Table S13: Ablation experiments of the GNN module on the PBMC dataset.


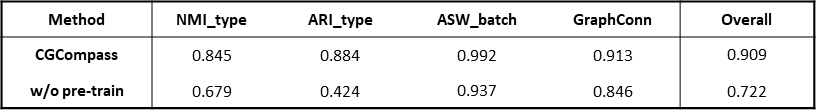


Table S14: Ablation experiments of the pre-training stage on the PBMC dataset.


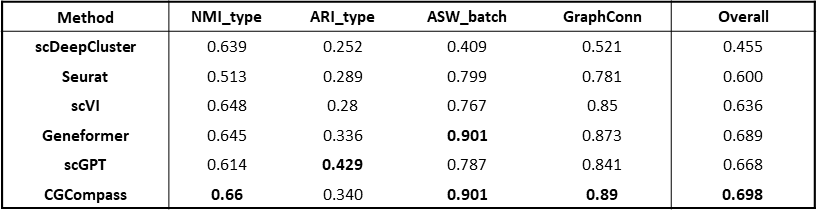
Table S15: Fine-tuning results of batch integration on the Covid dataset.


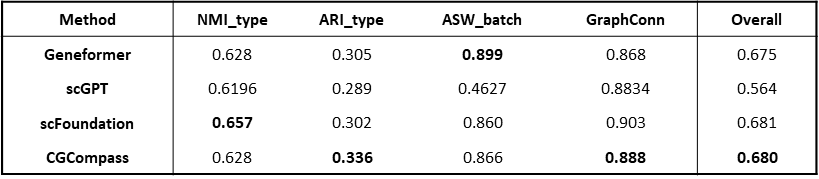
Table S16: Zero-shot results of batch integration on the Covid dataset.


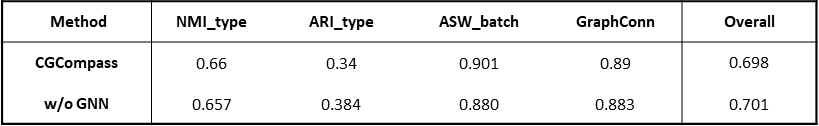
Table S17: Ablation experiments of the GNN module on the Covid dataset.


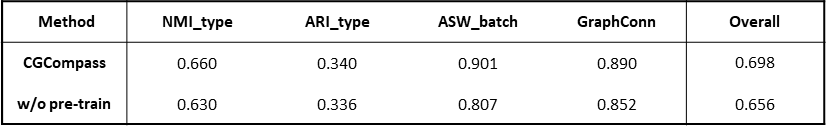
Table S18: Ablation experiments of the pre-training stage on the Covid dataset.


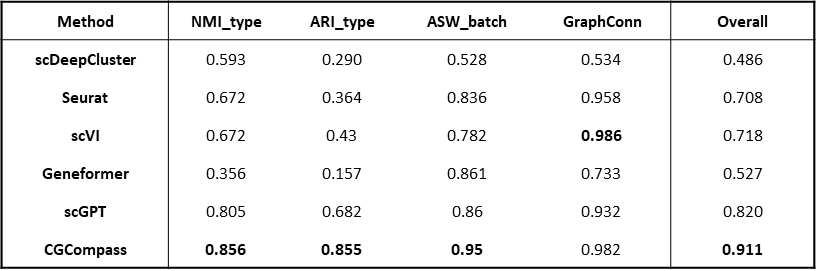
Table S19: Fine-tuning results of batch integration on the hPancreas dataset.


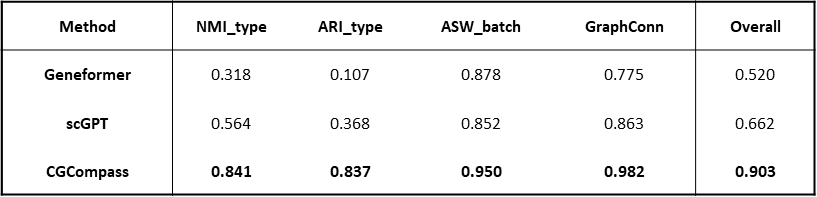


Table S20: Zero-shot results of batch integration on the hPancreas dataset.

**S.1.2 Cell type annotation**

We train the models on the reference set and test them on the query set. Accuracy is computed as the micro-average, while Precision, Recall, and F1-score are calculated as the macro-averages. The macro-average is based on the number of cell types in the reference set.


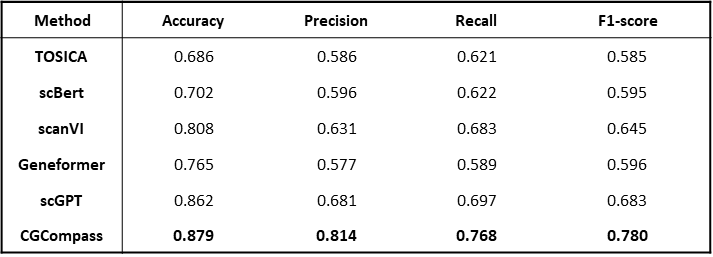


Table S21: Fine-tuning results of cell type annotation on the M.S. dataset.


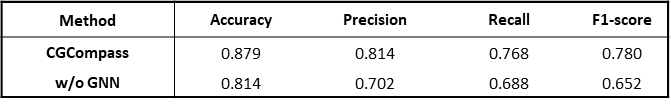


Table S22: Ablation experiments of the GNN module on the M.S. dataset.


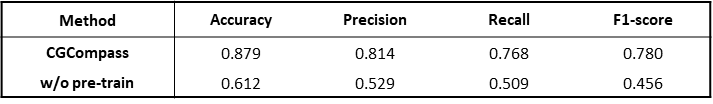


Table S23: Ablation experiments of the pre-training stage on the M.S. dataset.


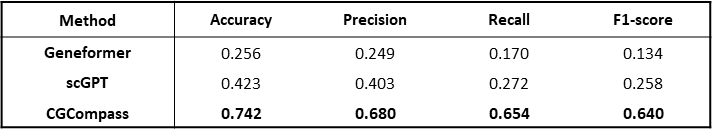


Table S24: Linear probing results of cell type annotation on the M.S. dataset.


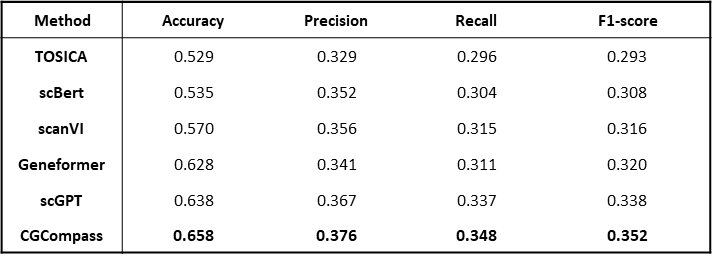


Table S25: Fine-tuning results of cell type annotation on the Mye. Dataset.


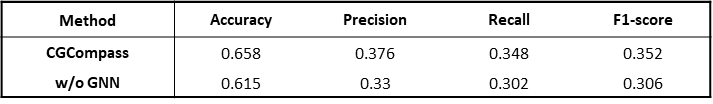


Table S26: Ablation experiments of the GNN module on the Mye. Dataset.


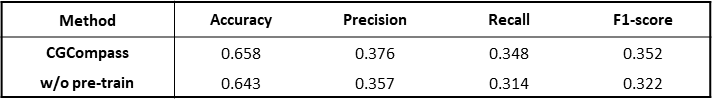


Table S27: Ablation experiments of the pre-training stage on the Mye. Dataset.


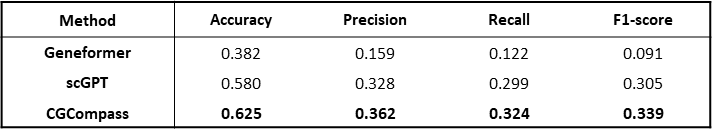


Table S28: Linear probing results of cell type annotation on the Mye. Dataset.


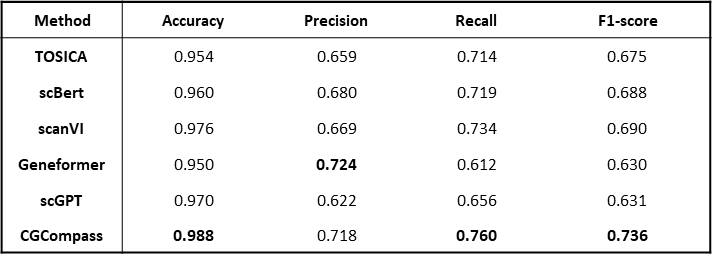


Table S29: Fine-tuning results of cell type annotation on the hPancreas dataset.


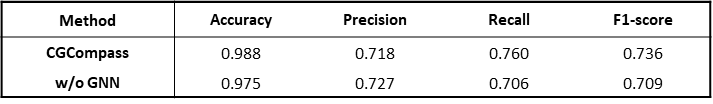


Table S30: Ablation experiments of the GNN module on the hPancreas dataset.


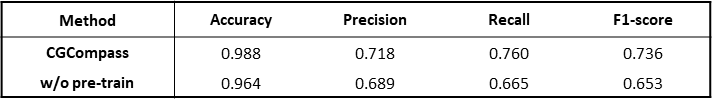


Table S31: Ablation experiments of the pre-training stage on the hPancreas dataset.


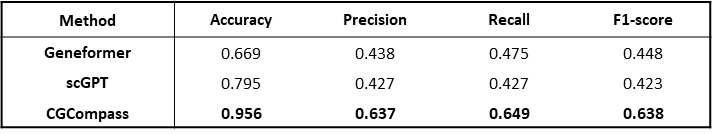


Table S32: Linear probing results of cell type annotation on the hPancreas dataset.


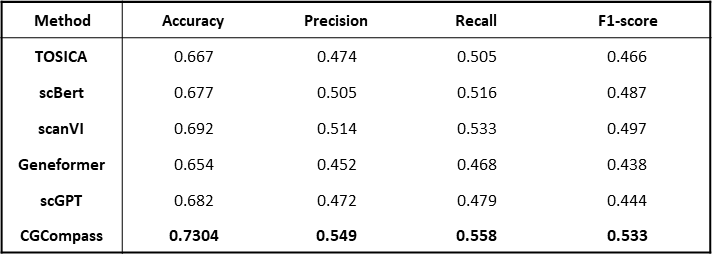


Table S33: Fine-tuning results of cell type annotation on the Lung dataset.


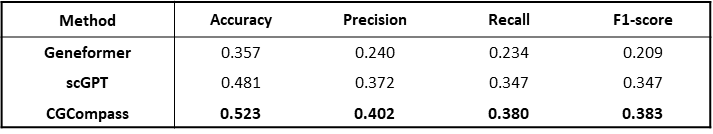


Table S34: Linear probing results of cell type annotation on the Lung dataset.

**S.1.3 Single-cell gene perturbation prediction**

We evaluate the performance of all models on three datasets. We first conducted prediction experiments at the single-cell resolution, followed by experiments at the pseudo-bulk level.


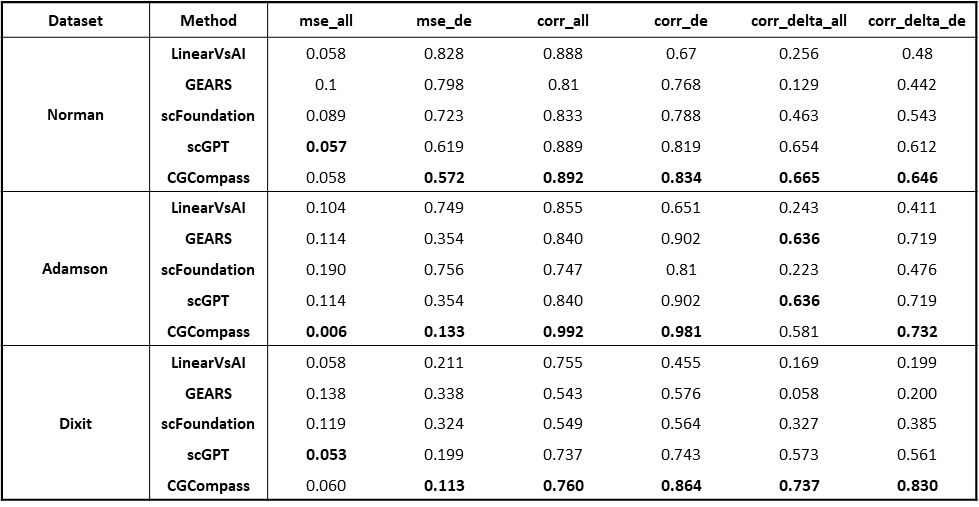
Table S35: Single-cell resolution perturbation response prediction. The *mse* and *corr* represent the mean squared error and Pearson correlation coefficient between the predicted and true gene expression profiles after perturbation. *corr_delta* refers to the correlation of the predicted change in expression. The suffix “*_all*” indicates evaluation across all genes, while “*_de*” refers to evaluation on the top 20 differentially expressed genes. *Norman* is a double-gene perturbation dataset, while the other two are single-gene perturbation datasets. *Dixit* is a small-sample dataset.


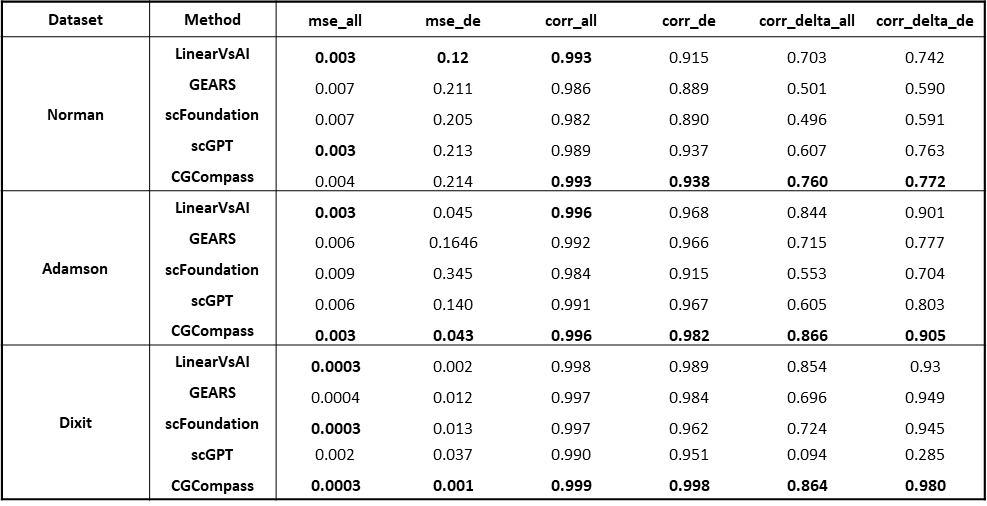


Table S36: Pseudo-bulk level perturbation response prediction.

**S.2 Benchmarking different foundation models on the same pre-training corpus**

We uniformly re-trained Geneformer, scGPT, and CGCompass on scCompass-h5M (scCompass-h50M with 10% random sampling), and then performed fine-tuning and comparison experiments on several datasets for each of the three tasks: batch integration, cell type annotation, and single-cell gene perturbation.

**
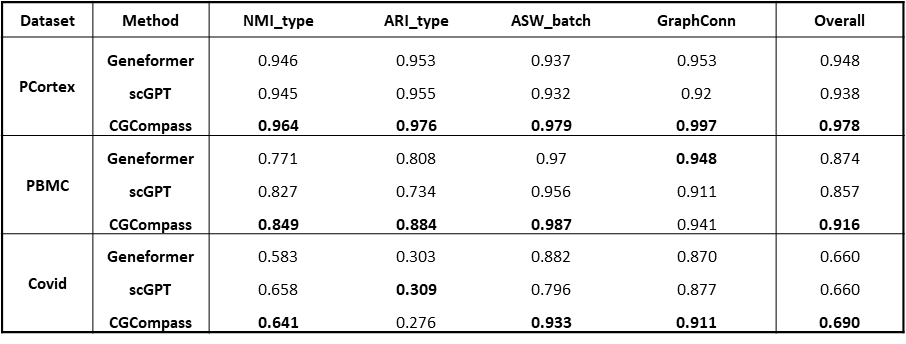
**

Table S37: Benchmark results on batch integration (all pre-trained on scCompass-h5M).

**
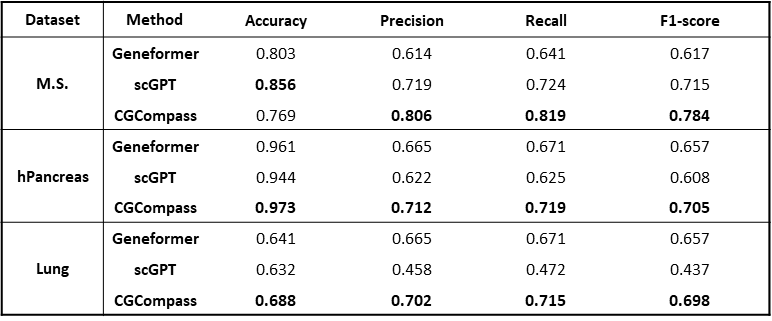
**

Table S38: Benchmark results on cell type annotation (all pre-trained on scCompass-h5M).

**
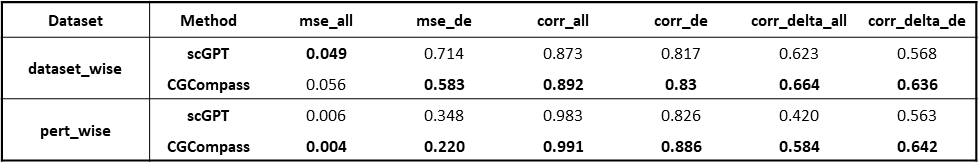
**

Table S39: Benchmark results of single-cell gene perturbation on the Norman dataset (all pre-trained on scCompass-h5M).

**
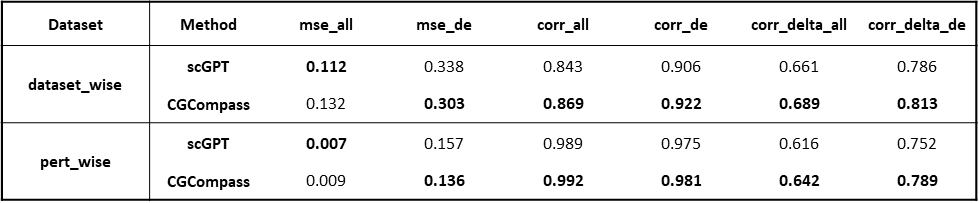
**

Table S40: Benchmark results of single-cell gene perturbation on the Adamson dataset (all pre-trained on scCompass-h5M).

**
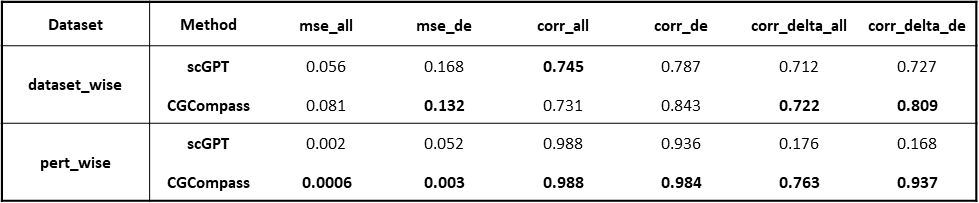
**

Table S41: Benchmark results of single-cell gene perturbation on the Dixit dataset (all pre-trained on scCompass-h5M).

**S.3** **Ablation experiments on different biological features used by CGCompass**

CGCompass constructs the cell graph using six biological features. Among these, gene tokens define the gene identities considered in modeling the cell, while gene expression values capture the context-aware information for the cell. Therefore, we systematically ablated the other four features, as well as all of them together, and conducted fine-tuning experiments for each task: batch integration, cell type annotation, and single-cell gene perturbation. All five ablation models were pre-trained on scCompass-h5M.

**
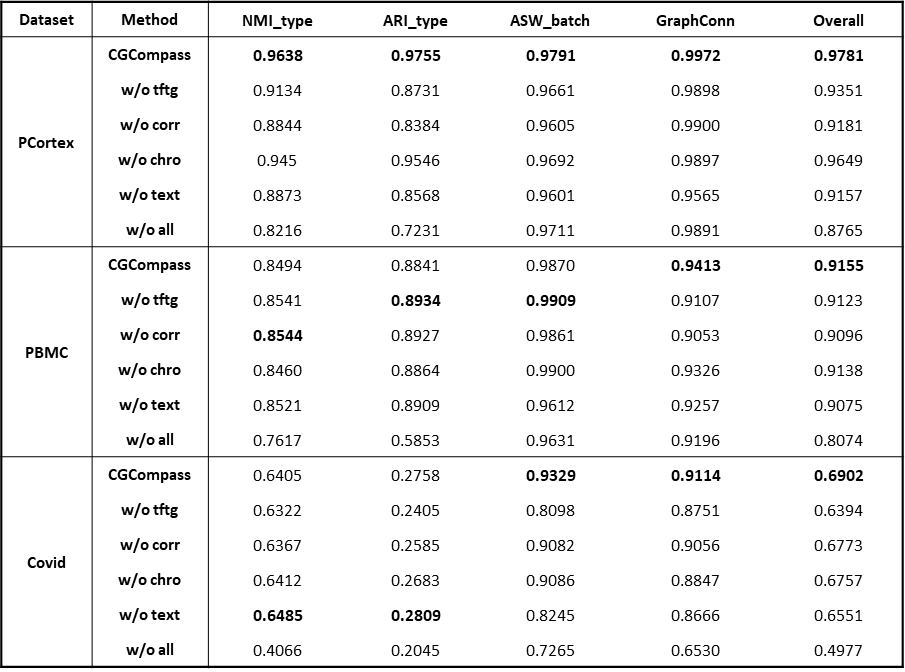
**

Table S42: Results of feature ablation on the batch integration task.

**
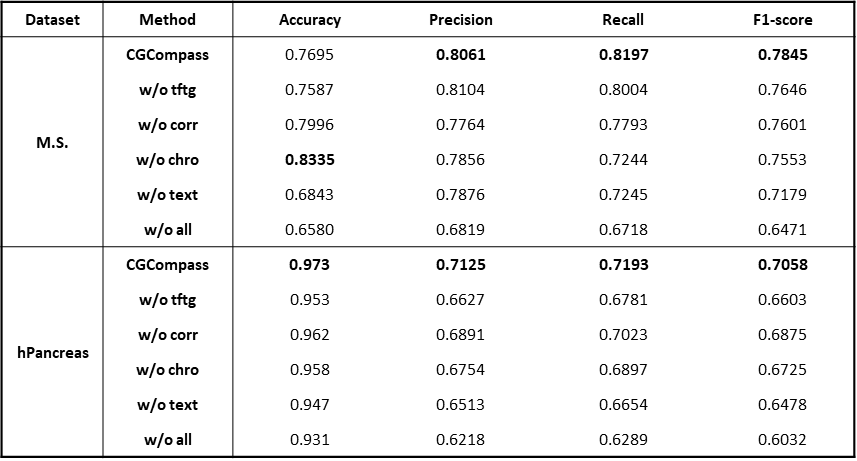
**

Table S43: Results of feature ablation on the cell type annotation task.


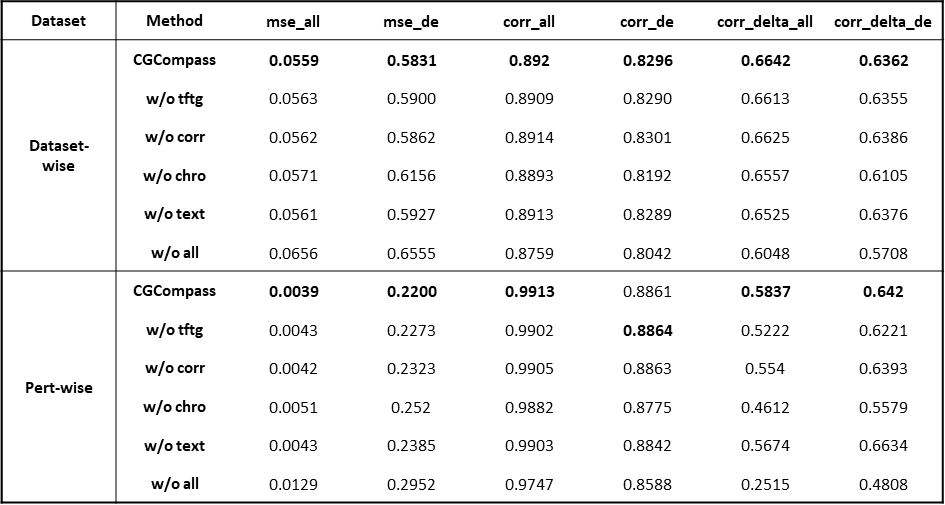
Table S44: Results of feature ablation on the single-cell perturbation dataset Norman.

**
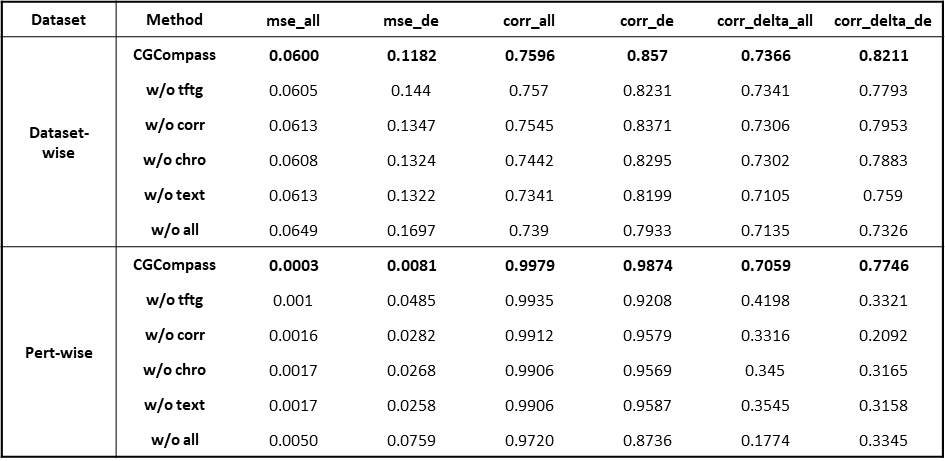
**

Table S45: Results of feature ablation on the single-cell perturbation dataset Dixit.

**S.4** **Computational resources used for the pretraining of CGCompass**

We recorded the memory usage and pretraining time for Geneformer, scGPT, and CGCompass on scCompass-h5M. All experiments were conducted on a single machine with six A100-40G GPUs. The pretraining ran for 5 epochs, with *Flash Attention* acceleration used for both scGPT and CGCompass.

**
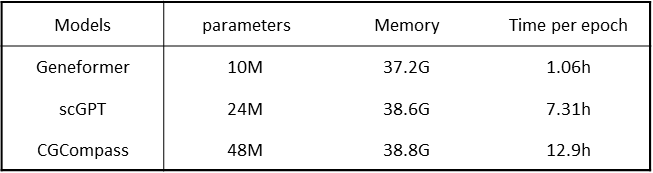
**

Table S46: Training time and memory usage for pretraining different foundation models on scCompass-h5M, conducted on six A100-40G GPUs.
